# Machine Learning Algorithms Versus Classical Regression Models in Pre-Eclampsia Prediction: A Systematic Review

**DOI:** 10.1007/s11906-024-01297-1

**Published:** 2024-05-28

**Authors:** Sofonyas Abebaw Tiruneh, Tra Thuan Thanh Vu, Daniel Lorber Rolnik, Helena J. Teede, Joanne Enticott

**Affiliations:** 1 Monash Centre for Health Research and Implementation, Faculty of Medicine, Nursing and Health Sciences, Monash University, Melbourne, Australia; 2 Department of Obstetrics and Gynaecology, Monash University, Clayton, VIC Australia

**Keywords:** Pre-eclampsia, Prediction models, Classical regression, Machine learning, Discrimination, Calibration

## Abstract

**Purpose of Review:**

Machine learning (ML) approaches are an emerging alternative for healthcare risk prediction. We aimed to synthesise the literature on ML and classical regression studies exploring potential prognostic factors and to compare prediction performance for pre-eclampsia.

**Recent Findings:**

From 9382 studies retrieved, 82 were included. Sixty-six publications exclusively reported eighty-four classical regression models to predict variable timing of onset of pre-eclampsia. Another six publications reported purely ML algorithms, whilst another 10 publications reported ML algorithms and classical regression models in the same sample with 8 of 10 findings that ML algorithms outperformed classical regression models. The most frequent prognostic factors were age, pre-pregnancy body mass index, chronic medical conditions, parity, prior history of pre-eclampsia, mean arterial pressure, uterine artery pulsatility index, placental growth factor, and pregnancy-associated plasma protein A. Top performing ML algorithms were random forest (area under the curve (AUC) = 0.94, 95% confidence interval (CI) 0.91–0.96) and extreme gradient boosting (AUC = 0.92, 95% CI 0.90–0.94). The competing risk model had similar performance (AUC = 0.92, 95% CI 0.91–0.92) compared with a neural network. Calibration performance was not reported in the majority of publications.

**Summary:**

ML algorithms had better performance compared to classical regression models in pre-eclampsia prediction. Random forest and boosting-type algorithms had the best prediction performance. Further research should focus on comparing ML algorithms to classical regression models using the same samples and evaluation metrics to gain insight into their performance. External validation of ML algorithms is warranted to gain insights into their generalisability.

**Supplementary Information:**

The online version contains supplementary material available at 10.1007/s11906-024-01297-1.

## Introduction

Pre-eclampsia is a multisystem disorder of pregnancy characterised by new onset of elevated blood pressure and proteinuria or hypertension and significant end-organ dysfunction with or without proteinuria after 20 weeks of gestation or postpartum in previously normotensive women [1, 2]. Pre-eclampsia affects 2–8% of pregnancies worldwide and causes 76,000 maternal and 500,000 perinatal deaths each year [3–5].

Administration of low-dose aspirin in women with at high risk of pre-eclampsia before 16-week gestation has been shown to reduce the risk of pre-eclampsia and adverse perinatal health outcomes [6–9]. Clinical risk prediction models are used in healthcare to identify those at risk and to guide diagnosis, prevention, and prognosis [10]. These use readily available data, such as demographic information, clinical characteristics [11–13], and specialised biomarkers [14, 15]. Maternal medical and clinical characteristics are the most used prognostic factors [11–13] that have the advantage of being widely available in non-specialised and low-resource settings; however, the addition of specialised biomarkers can improve prediction performance but might limit the implementation into low-resource settings [16].

Risk prediction models can be developed and validated either by applying classical regression models (for example, logistic regression, competing risk models) or machine learning (ML) algorithms (for example, decision tree, random forest, gradient boosting, and neural networks) [10, 17]. Classical regression prediction models are abundantly reported in the medical literature [18–21], whilst ML prediction algorithms are gaining in popularity in the field [22–24]. Differences between classical regression prediction model and ML algorithm approaches have been extensively discussed in the literature [25, 26]. Classical regression models are based on theory and assumptions [17]. In contrast, ML algorithms learn from the data with the ability to analyse non-linear data structures using fewer assumptions and modelling high dimensional data [27, 28]. Some studies report that ML algorithms manage more predictors and outperform classical regression models [29–31]; yet, others report no prediction performance advantage of ML algorithms [32, 33] in healthcare prediction models.

Previous systematic reviews and meta-analyses have investigated prediction performance based on classical regression models in pre-eclampsia prediction [34–36]. Currently, no systematic review has been conducted comparing the prediction performance of ML algorithms to classical regression models in pre-eclampsia prediction. This review aims to (1) explore the existing ML algorithms, classical regression prediction models, and potential prognostic factors in pre-eclampsia prediction and (2) compare the prediction performance of ML algorithms to that of classical regression models in pre-eclampsia prediction.

## Methods

### Search Strategies

This systematic review was conducted following the Preferred Reporting Items for Systematic Reviews and Meta-analyses (PRISMA) guideline [37]. We used the Population (pregnant women), Index prognostic model (developed prognostic models), Comparator (machine learning algorithms with classical regression models), Outcome (pre-eclampsia), Timing (prediction of pre-eclampsia after 20 weeks of gestation), and Setting (individualised risk stratification) PICOTS framework [38]. Pre-eclampsia is classified based on the gestational age at clinical presentation as any-onset (delivery at any gestation), preterm (delivery < 37 weeks of gestation), late-onset (delivery ≥ 34 weeks of gestation), and early-onset (delivery < 34 weeks of gestation) [39]. This review was registered with the International Prospective Register of Systematic Reviews (PROSPERO CRD42023445732).

Literature search was conducted on Ovid platform (MEDLINE, Embase, Emcare, and Maternity & Infant Database (MIDIRS)) and CINAHL databases. The search was conducted until 20 May 2023 without restriction of publication years. In addition, a Google Scholar grey literature search was conducted as per Enticott et al. (2018) [40]. We included studies from previously published systematic reviews which considered only classical regression models [34, 36]. The search strategies were developed following search filters for prediction and diagnostic studies [41] and in consultation with a university librarian. Medical Subject Heading (MeSH) terms and free text words were used to locate potential prediction models. Boolean operators (AND, OR, and NOT) and truncation were used to combine the search key terms. A detailed description of search combinations and strategies is given in Supplementary File 1.

### Eligibility Identification

Prediction models for pre-eclampsia (any-, early-, and late-onset and preterm) conducted using cohort/follow-up, nested case–control, case–control, case-cohort, randomised controlled trial, and routinely collected health records data sources were included in this review. We excluded prediction model studies focused exclusively on hypertensive disorders of pregnancy or gestational hypertension unless they also provided a distinct model for pre-eclampsia. Studies conducted on selected populations (only twin pregnancies, only high-risk/low-risk women), studies in languages other than English, and prognostic studies conducted with only single prognostic factors were excluded from this review. Furthermore, external validation prediction studies were excluded from the comparison.

### Screening and Methodological Quality Appraisal

The included studies were screened using the Covidence platform [42]. After duplicates were removed, two authors (SAT and TV) independently assessed the title and abstract followed by full-text screening. Discrepancies between the two authors were resolved through discussion.

### Assessment of Methodological Quality for Classical Regression Models

The risk of bias (ROB) and concern for applicability [43] was assessed using the Prediction model Risk Of Bias ASsessment Tool (PROBAST) tool by two authors (SAT and TV). The tool has four domains (participants, predictors, outcomes, and analysis) structured into 20 signalling questions. Each included study rated as high, low, or unclear risk of bias for both ROB and concern for applicability.

### Data Extraction

The CHecklist for critical Appraisal and data extraction in systematic Reviews of clinical prediction Modelling Studies (CHARMS) tool was used to extract the data [44]. Authors, publication year, country, data sources, outcome(s) to be predicted, candidate prognostic factors, sample size, type of models or algorithms, internal validation methods, discrimination performance, and calibration measures were extracted. The algorithm’s discrimination and calibration performance were extracted from the test dataset for studies that specifically conducted internal validations; otherwise, from the development dataset. The model/algorithm deployment strategy was also extracted. Deployment strategies, such as regression formulae, nomograms, and score chart rules, are methods used to employ an algorithm/model into a system, enabling it to predict outcomes for new clients. Two authors (SAT and TV) independently extracted the data. Disagreements were managed through discussion and by another author (JE) if necessary.

### Data Analysis

The descriptive synthesis was performed for both ML and classical regression studies. Prognostic factors were identified. Algorithm/model discrimination and calibration performance were narratively described and compared. ML algorithms and classical regression model prediction performance were primarily compared in studies that used the same sample. Furthermore, the prediction performance was compared across overall ML algorithms and classical regression models. The discrimination performance for studies reporting on both ML and classical regression models was visualised in a forest plot so that readers can easily compare the performances. Model discrimination refers to the model ability to correctly classify and discriminate between participants who had the outcome of interest and those who did not, often measured by the area under the receiver-operating characteristics (ROC) curve. An area under the curve (AUC) value = 0.5 suggests no discrimination ability, 0.5 < AUC < 0.7 is considered as poor discrimination, 0.7 ≤ AUC < 0.8 is good/acceptable discrimination, 0.8 ≤ AUC < 0.9 is excellent discrimination, and AUC ≥ 0.9 is considered outstanding discrimination performance [45]. Calibration reflects how well the predicted risks match the observed risks of an outcome of interest. This is often measured by comparing the mean predicted probability and the observed outcome rates within risk groups and by the Hosmer and Lemeshow statistic. A well-calibrated model is when the Hosmer–Lemeshow *p* value is not significant and/or the calibration slope value approaches one and/or calibration-in-the-large close to zero [46, 47].

## Results

### Study Selection and Search Strategies

**Fig. 1:**
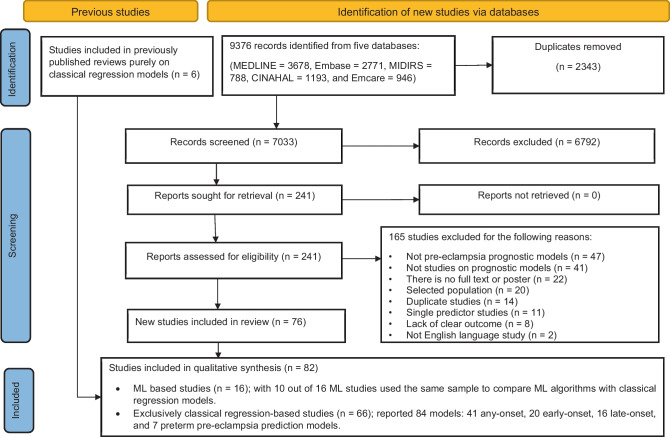
PRISMA flow diagram for the inclusion and exclusion criteria [37]

We retrieved 9376 records from five electronic databases and an additional six studies from previously published systematic reviews which considered only classical regression models [34, 36]. After 2343 duplicates were removed, 7033 articles were excluded through title and abstract screening, leaving 241 articles eligible for full-text review. In the full-text screening, 76 records met inclusion criteria. Finally, based on the database search and previously published systematic reviews of classical regression models, we included 82 developed studies (ten with both ML algorithm and classical regression models, six with ML only, and 66 with classical regression only) (Fig. 1).

### Characteristics of ML-Based Prediction Studies

Table 1 shows that sixteen [48•, 49•, 50–57, 56, 59•, 60•, 61–63] (fourteen any-onset and two preterm pre-eclampsia) ML studies were included and reported from 2019 to 2023. Ten studies reported both ML algorithms and classical regression models. Four ML studies were developed in China [48•, 49•, 50, 61], two in the United States of America (USA) [51, 52], two in Romania [53, 59•], and the rest were from United Kingdom [60•], Indonesia [54], New Zealand [55], Slovenia [56], South Korea [57], Sweden [58], Kenya [62], and Iran [63]. Case–control, retrospective/prospective cohort, and medical record data sources were used in the included studies. The maximum sample size was 60,789, the minimum was 95, and one [53] study did not report the sample size and/or event rate. Decision tree, naïve Bayes, support vector machine, random forest, gradient boosting machine, extreme gradient boosting machine (XGBoost), light boosting, neural network, Viterbi ML, and classification via regression ML algorithms were reported (Table 1).

**Table 1:** Characteristics of ML algorithm prediction studies

S. No	Studies	Country	Data source	Centre	Outcome	Events/sample size (events per predictor)	Best performing algorithm
1	Melinte-Popescu et al. 2023 [59•]	Romania	Case–control	Single	Any-onset	116/233 (17)	Random forest
2*	Liu et al. 2022 [48•]	China	Retrospective cohort	Single	Any-onset	143/11,152 (14)	Random forest
3	Zhang et al. 2022 [50]	China	Retrospective cohort	Single	Any-onset	377/19,653 (126)	Light GBM
4	Gómez-Jemes et al. 2022 [56]	Slovenia	Medical record	Single	Any-onset	22/95 (7)	Decision tree
5	Bennett et al. 2022 [52]	USA	Prospective cohort	Multicentre	Any-onset	2743/31,431 (137)	Deep neural networks
6*	Ansbacher-Feldman et al. 2022 [60•]	UK	Prospective cohort	Multicentre	Preterm	484/60789 (35)	Neural network
7*	Chen et al. 2022 [61]	China	Case–control	Single	Any-onset	237/916 (40)	Random forest
8*	Li et al. 2021 [49•]	China	Retrospective cohort	Single	Any-onset	227/5243 (76)	XGBoost
9*	Wanriko et al. 2021 [62]	Kenya	Case–control	Single	Any-onset	88/352 (7)	Random forest
10*	Manoochehri et al. 2021 [63]	Iran	Case–control	Single	Any-onset	752/1452 (125)	SVM
11*	Marić et al. 2020 [51]	USA	Retrospective cohort	Single	Any-onset	561/5245 (80)	Gradient boosting
12*	Sufriyana et al. 2020a [54]	Indonesia	Nested case–control	Single	Any-onset	878/6734 (58)	Random forest
13	Sufriyana et al. 2020b [55]	New Zealand	Prospective cohort	Single	Any-onset	22/95 (4)	CVR
14	Marin et al. 2019 [53]	Romania	Medical record	Single	Any-onset	NR	Viterbi ML
15*	Jhee et al. 2019 [57]	South Korea	Medical record	Single	Any-onset	474/10,532 (67)	Gradient boosting
16*	Sandström et al. 2019 [58]	Sweden	Prospective cohort	Single	Preterm	497/58,276 (41)	Logistic regression

NB: *XGBoost* extreme gradient boosting, *CVR* classification via regression, *SVM* support vector machine, *NR* not reported

^*^The studies that used the same sample to compare ML algorithms with classical regression models

### Distribution of Prognostic Factors

**Fig. 2:**
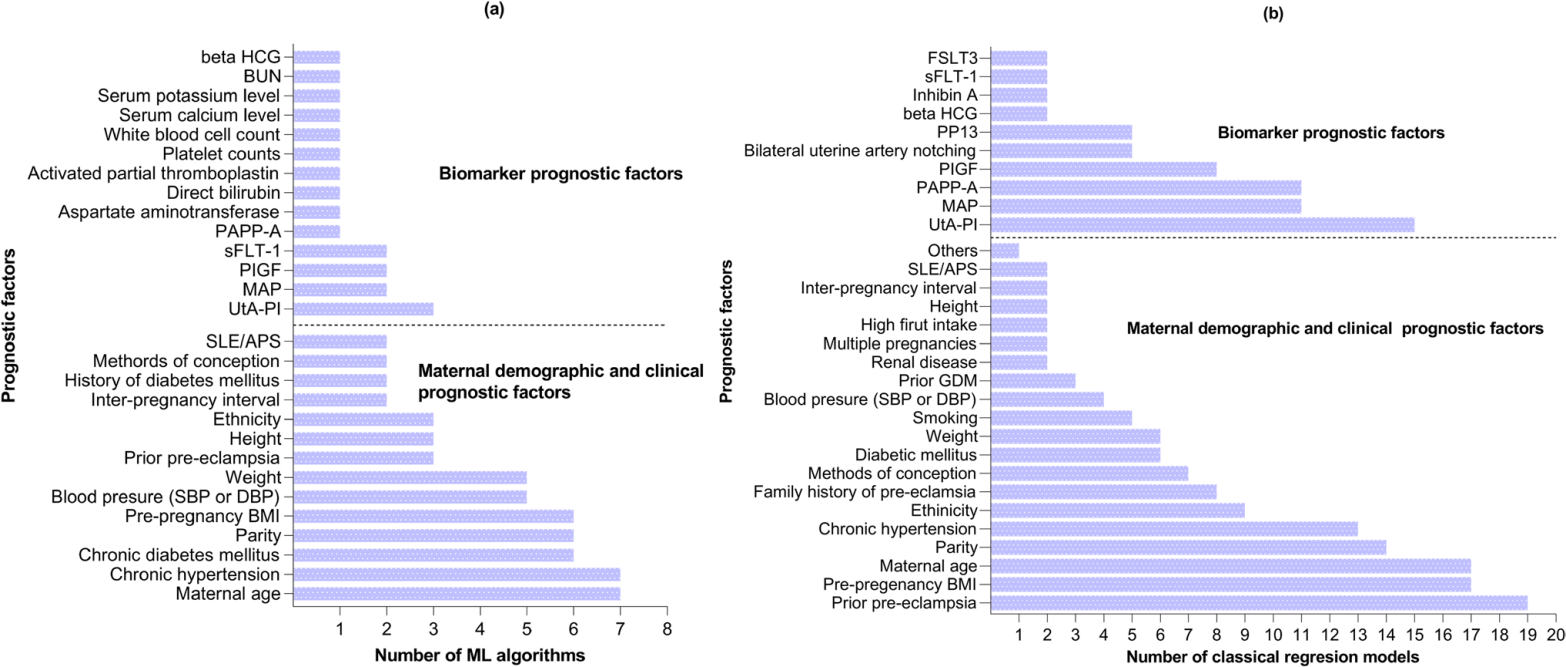
Distribution of prognostic factors across ML algorithms (**a**) and classical regression-based models (**b**). For the 16 ML algorithms, the number of feature variables ranged from 3 to 17, with a median of 7. For the 41 any-onset pre-eclampsia classical regression models, the number of predictor variables ranged from 2 to 13, with a median of 5. In the ten studies with both ML algorithm and classical regression model, the number of feature variables ranged from 3 to 17, with a median of 8. NB: Others = alcohol consumption in the first trimester, family history of chronic heart disease, and single miscarriage

Maternal demographic, medical, and clinical factors and a variety of biomarkers were commonly included in ML and classical regression studies to predict pre-eclampsia. Figure 2a shows the distribution of prognostic factors used in ML studies. Maternal age, chronic hypertension and diabetes mellitus, parity/gravidity, pre-pregnancy body mass index (BMI), blood pressure measurements, weight, prior history of pre-eclampsia, and ethnicity were the most frequently used maternal medical and clinical prognostic factors in ML studies. Uterine artery pulsatility index (UtA-PI) was the most frequently used biomarker in ML studies. Figure 2b shows the distribution of prognostic factors used in classical regression models. Family history of pre-eclampsia, prior history of pre-eclampsia, pre-pregnancy BMI, parity, chronic hypertension, and ethnicity were the most frequently used prognostic factors in classical regression models. Uterine artery pulsatility index (UtA-PI), mean arterial pressure (MAP), pregnancy-associated plasma protein A (PAPP-A), and placental growth factor (PIGF) were the most frequently used biomarkers in classical regression models (Fig. 2).

### ML Algorithm Performance and Comparison with Classical Regression Models

**Fig. 3:**
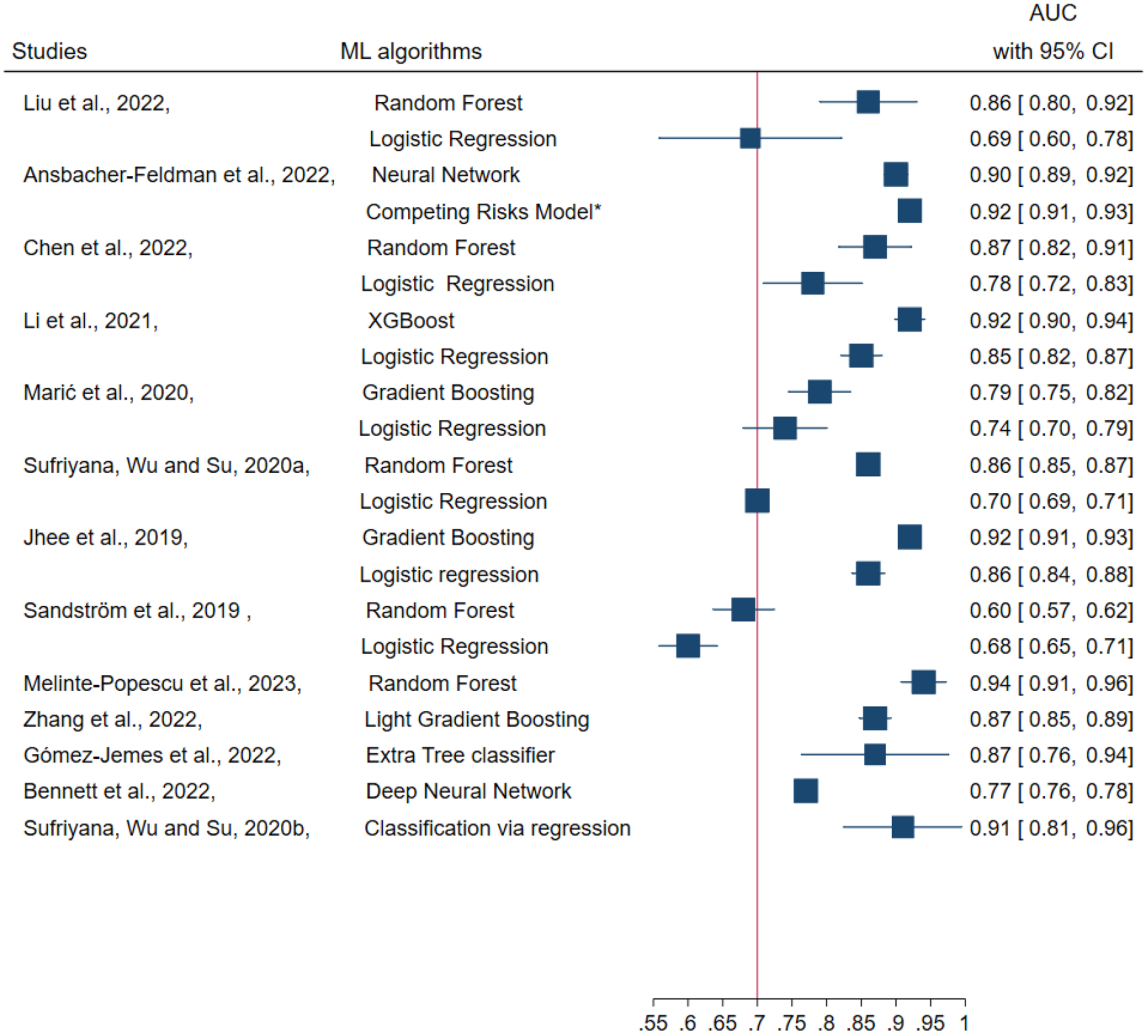
Machine learning algorithm performance (reported in 13/16 studies). Among the ten studies that reported both ML algorithms and classical regression models, the top eight reported discrimination performance (AUC), and the remaining did not. NB: The red vertical line highlights algorithms/models with AUC cut-off values above 0.7, which indicates good discrimination performance. *This classical regression model used the same sample as the ML algorithm above it and was reported in a separate publication [19]

Figure 3 shows model discrimination performance of thirteen ML studies. Three [53, 62, 63] ML studies did not report model discrimination performance through AUC values. Ten studies [48•, 49•, 51, 54, 57, 58, 60•, 61–63] reported both ML algorithms and classical regression model performance; eight studies [48•, 49•, 51, 54, 57, 58, 61–63] reported that ML algorithms have better prediction performance than classical regression models. Another study [60•] showed that there is no difference in prediction performance between competing risks preterm pre-eclampsia model and ML algorithms. Only one preterm pre-eclampsia [58] prediction model used logistic regression showed better prediction performance than a random forest algorithm. The minimum AUC of ML algorithms was 0.60 (95% CI 0.57–0.62) and the maximum AUC was 0.94 (95% CI 0.91–0.96). Two studies [62, 63] have not reported algorithm/model discrimination (AUC) performance, however reported prediction accuracy. Overall, random forest and boosting-type algorithms (gradient boosting and XGBoost) showed better prediction performance than other ML algorithms (Fig. 3). Three [48•, 49•, 55] models were well-calibrated, one [54] model was not well-calibrated, and the rest studies did not report model calibration performance. Except two [53, 59•], ten ML studies reported split sample, and four studies reported cross-validation for internal validation; yet none reported external validation. None of the ML studies provided deployment strategies for individualised risk prediction (Supplementary. Table 1).

### Characteristics of Classical Regression-Based Prediction Studies

Sixty-six publications [14, 64, 73–82, 65, 83–92, 66, 93–102, 67, 103–112,68, 113–122, 69, 123–128,70–72] reporting on 84 models were included:

- Forty-one any-onset pre-eclampsia models (Table 2)
- Twenty early-onset pre-eclampsia models (Supplementary Table 3)
- Sixteen late-onset pre-eclampsia models (Supplementary Table 4)
- Seven preterm pre-eclampsia models (Supplementary Table 5)

**Table 2:** Characteristics of classical regression models for any-onset pre-eclampsia prediction

S. No	Author, year	Country	Data sources	Centre	Events/sample size (events per predictors)
1	Suksai et al. 2022 [65]	Thailand	Routinely collected	Single	167/4600 (14)
2	Tarca et al. 2022 [66]	USA	Case cohort	Single	166/1150 (42)
3	Tang et al. 2022 [67]	China	Retrospective cohort	Single	717/20,582 (102)
4	Yue et al. 2021 [68]	China	Routinely collected	Single	310/6064 (34)
5	Kim et al. 2021 [69]	China	Prospective cohort	Single	13/351 (4)
6	Wang et al. 2020 [70]	China	Prospective cohort	Single	25/356 (6)
7	Al-Rubaie et al. 2020 [71]	Australia	Retrospective cohort	Multicentre	293/12,395 (37)
8	Sovio and Smith 2019 [72]	UK	Prospective cohort	Single	28/4184 (3)
9	Boutin et al. 2019 [73]	Canada	Prospective cohort	Single	232/4739 (38)
10	Boutin et al. 2018 [74]	Canada	Prospective cohort	Single	232/4665 (33)
11	Cheng et al. 2018 [75]	China	Case–control	Single	30/3330 (10)
12	Praciano de Souza et al. 2018 [76]	Brazil	Prospective cohort	Single	40/372 (10)
13	Asiltas et al. 2018 [77]	Turkey	Case–control	Single	38/160 (12)
14	Rocha et al. 2017 [78]	Brazil	Prospective cohort	Multicentre	55/733 (14)
15	Luo and Han 2017 [79]	China	Case–control	Single	33/104 (11)
16	Agarwal et al. 2017 [80]	India	Nested case–control	Single	35/291(11)
17	Guy et al. 2017 [81]	UK	Prospective cohort	Multicentre	66/2764 (5)
18	Gabbay‐Benziv et al. 2016 [82]	USA	Prospective cohort	Single	108/2433 (21)
19	Kumar et al. 2016 [83]	India	Prospective cohort	Single	98/3069 (20)
20	Giguere et al. 2015 [84]	Canada	Nested case–control	Single	96/343 (16)
21	Wright et al. 2015 [85]	UK	Prospective cohort	Multicentre	2704/120,492 (540)
22	Moon and Odibo 2015 [86]	USA	Prospective cohort	Single	102/1177 (15)
23	Baschat et al. 2014 [87]	USA	Prospective cohort	Multicentre	108/2441 (27)
24	Kenny et al. 2014 [88]	SCOPE^a^	Prospective cohort	Multicentre	278/5623 (56)
25	Goetzinger et al. 2014 [89]	USA	Prospective cohort	Single	49/578 (8)
26	Gurgel Alves et al. 2014 [90]	Brazil	Prospective cohort	Single	31/550 (6)
27	Teixeira et al. 2014 [91]	Portugal	Retrospective cohort	Single	140/4799 (12)
28	Skråstad et al. 2014 [92]	Norway	Prospective cohort	Single	39/640 (10)
29	Direkvand-Moghadam et al. 2013 [93]	Iran	Prospective cohort	Single	58/610 (11)
30	North et al. 2011 [94]	SCOPE^a^	Prospective cohort	Multicentre	186/3529 (16)
31	Odibo et al. 2011 [95]	USA	Prospective cohort	Single	42/452 (14)
32	Yu et al. 2011 [96]	China	Case–control	Single	31/124 (7)
33	Goetzinger et al. 2010 [97]	USA	Retrospective cohort	Single	293/3716 (59)
34	Thilaganathan et al. 2010 [98]	UK	Nested case–control	Single	45/170 (15)
35	Poon et al. 2008 [99]	UK	Prospective cohort	Single	104/5193 (26)
36	Deis et al. 2008 [100]	France	Prospective cohort	Single	110/4777 (18)
37	De Paco et al. 2008 [101]	UK	Prospective cohort	Single	83/4617 (17)
38	Pilalis et al. 2007 [102]	Greece	Prospective cohort	Single	13/878 (2)
39	Yu et al. 2005 [103]	UK	Prospective cohort	Multicentre	612/30,708 (76)
40	Papageorghiou et al. 2005 [104]	UK	Prospective cohort	Multicentre	369/17,480 (53)
41	Harrington et al. 1997 [105]	UK	Prospective cohort	Single	30/626 (7)

NB: ^a^New Zealand, Australia, the UK, and Ireland

### Any-Onset Pre-Eclampsia Models

Forty-one [65, 66, 75–84, 67, 85–94, 68, 95–104, 69, 105, 70–74] any-onset pre-eclampsia prediction models were included. The maximum sample size was 120,492, and the minimum sample size was 104 for any-onset pre-eclampsia prediction models. Almost all (40/41) any-onset pre-eclampsia prediction models were from middle or high-income countries. Nine pre-eclampsia prediction models were reported from the United Kingdom (UK) [72, 81, 85, 98, 99, 101, 103–105], six from the USA [66, 82, 86, 87, 89, 97], six from China [67–70, 79, 96], three from Brazil [76, 78, 90], three from Canada [73, 74, 84], two from the SCOPE study [88, 94], and one each from Australia [71], Thailand [65], Turkey [77], India [83], Iran [93], Greece [102], France [100], Australia [71], Norway [92], and Portugal [91]. Twenty-four (10/42) percent of any onset pre-eclampsia prediction models were developed with less than ten events per prognostic factor (Table 2). Only fifteen any-onset pre-eclampsia prognostic models report the model equation. Among any-onset pre-eclampsia prediction models, ten studies reported regression formulae and eleven reported nomogram and score chart rule for deployment strategy to estimate individualised risks. The remaining models did not report deployment strategies (Supplementary Table 2).

### Early-Onset Pre-Eclampsia Models

Twenty [67, 83, 113–122, 87, 106–112] early-onset pre-eclampsia models were included in this review. The maximum [115] sample size reported was 33,602, and the minimum [111] sample size was 359. Ninety percent of the studies were from middle and high-income countries. Six studies were from the UK [115, 117–120, 122], three from France [107, 109, 110], two from the Netherlands [112, 116], two from Chile [111, 113], and one each from China [67], Spain [106], India [83], Finland [108], the USA [87], Italy [114], and Denmark [121]. Only five [106, 107, 112, 115, 121] developed models had more than ten events per prognostic factor (Supplementary Table 3).

### Late-Onset Pre-Eclampsia Models

Sixteen [64, 83, 118–123, 107, 109–112, 114, 115, 117] late-onset pre-eclampsia prediction models were included. The maximum sample size was reported 33,602, and 359 was the minimum sample size. Eighty-eight percent (14/16) of the models reported were from high-income countries. Six models were developed in the UK [115, 117–120, 122], three models in France [107, 109, 110], two models in Italy [114, 123], and one each from India [83], Thailand [64], Chile [111], and Denmark [121]. Sixty-nine percent of models used more than ten events per predictor (Supplementary Table 4).

### Preterm Pre-Eclampsia Models

Seven preterm [14, 67, 124–128] pre-eclampsia prediction models were included. Two models were from the UK [14, 128], one was multicentre international (SCOPE [127] study), and the other studies were one each from Sweden [124], China [67], Denmark [125], and Chile [126]. Only one model used less than ten predictor variables per event (Supplementary Table 5).

### Classical Regression Studies Prediction Performance

Almost all any-onset pre-eclampsia models reported discrimination performance but not model calibration. Ninety percent of any-onset pre-eclampsia models (36/40) reported good discrimination performance (AUC > 0.70). The minimum AUC reported was 0.62 (0.58–0.66) [74] and the maximum AUC reported was 0.96 (0.92–1) [80]. Calibration performance was not reported in most studies. Only four models [68, 71, 94, 100] reported calibration performance, and three were well-calibrated. Fifteen models reported deployment strategies. Ninety percent (18/20) of the early-onset pre-eclampsia prediction models have reported the model discrimination performance, whereas one study [116] has reported calibration performance. Ninety-four percent of the studies showed excellent to perfect discrimination performance, with a minimum AUC of 0.78 [67] and maximum AUC of 0.99 (0.99–1) [122]. The deployment of individualised risk stratification was reported in fourteen out of twenty early-onset pre-eclampsia models. Moreover, eleven out of sixteen late-onset pre-eclampsia prediction models have reported model discrimination performance and none of the studies report model calibration performance. Only five out of seven preterm pre-eclampsia prediction studies reported model discrimination performance with none of them reporting calibration performance. Most of the classical regression models failed to report internal validation, and nearly one-third (30/84) of the models were externally validated (Supplementary Table 6).

### Methodological Quality of ML and Classical Regression Studies

**Fig. 4:**
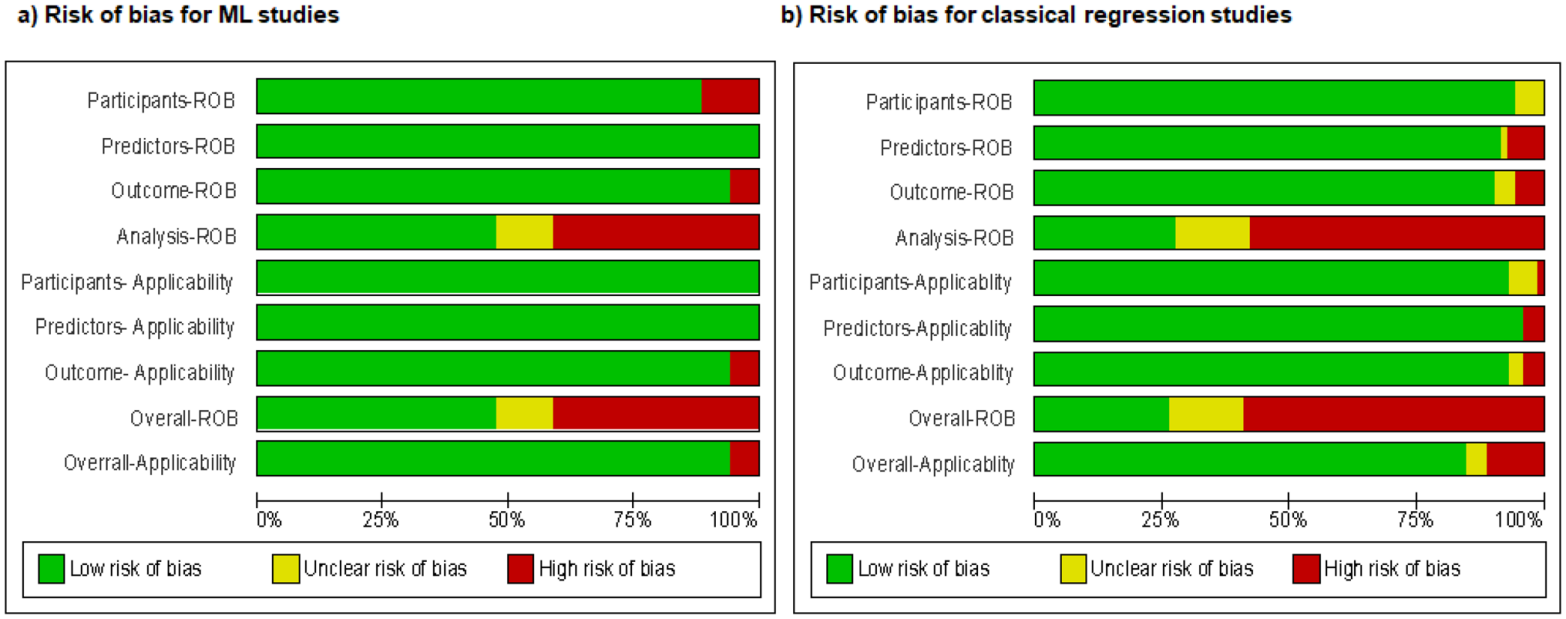
Risk of bias graph: review authors’ judgements about each risk of bias item presented as percentages across all included studies

Figure 4a shows the assessment of risk of bias (ROB) and concerns for applicability of ML studies. Overall, more than 40% of the ML studies have high risk of bias. Among four domains, the analysis domain had high risk of bias. Among studies at low risk of bias, discrimination performance (AUC) ranged from 0.77 to 0.92. Ninety-five percent of ML studies have low risk of concern for applicability. Figure 4b shows the ROB and concern for applicability of classical regression studies. Sixty percent of classical regression studies exhibited a high risk of bias with the analysis domain being the primary contributor. Among studies at low risk of bias, the AUC ranged from 0.66 to 0.89. More than 90% of classical regression studies have low risk of concern for applicability (Fig. 4).

## Discussion

Machine learning algorithm approaches are increasingly common in risk prediction [129, 130]; however, prediction performance compared with classical regression models remains unclear, including in pre-eclampsia prediction. This review identified 16 ML algorithms and 84 classical regression models for pre-eclampsia prediction, and overall, the ML approaches had the better prediction performance compared to the classical regression approaches. In the 10 studies reporting both ML algorithms and classical regression models in the same sample, eight [47, 48•, 50, 53, 56, 60•, 61–63] reported superior prediction performance for ML algorithms. The most frequent prognostic factors in all models were maternal demographic and clinical characteristics in pre-eclampsia prediction, with biophysical (UtA-PI, MAP) and biochemical (PAPP-A, PIGF) measurement being the most common biomarkers as prognostic factors. Almost all ML studies had reported internal validation, but failed to report external validation. All except three ML algorithms [52, 61, 63] reported discrimination performance with AUC ranging from 0.60 (95% CI 0.57–0.62) [57] to 0.94 (95% CI 0.91–0.96) [58]. Random forest, gradient boosting, and extreme gradient boosting algorithms were the top-performing ML algorithms. Of 66 classical regression studies reporting 84 models for any-, early-, late-onset, and preterm pre-eclampsia prediction showing poor-to-perfect discrimination performance, most failed to report model calibration. A high or unclear methodological risk of bias, yet low concern for applicability, was seen in both ML and classical regression studies. Deployment strategies were seen in some classical regression models, but not in ML algorithms.

Medical and clinical characteristics of the mother are the most cited risk factors for pre-eclampsia [11, 13]; similarly, we found these to be the most used prognostic factors in both ML and classical regression models. In addition, biomarker prognostic factors such as UtA-PI, MAP, PAPP-A, and PIGF were most frequently used in classical regression models whilst UtA-PI was most frequently used in ML algorithms, which is aligned to previous studies [115, 120]. The risk of pre-eclampsia can increase by eight-fold with prior pre-eclampsia history, seven-fold with obese pre-pregnancy BMI, five-fold with chronic hypertension, four-fold with chronic diabetes, three-fold in nulliparous woman, and a first-degree relative with pre-eclampsia [13]. Hence, the most frequently used prognostic factors in our review, in line with existing literature, but here combined in ML and classical regression models, have stronger predictive performance than when used in isolation. Considering only maternal medical and clinical characteristics have the advantages of readily attainable, easy to implement in all clinical settings, and cost-effective, however, addition of biomarkers could improve the prediction performance [15]. Machine learning prediction approach has the advantage of using raw biomarker data without the need for conversion into multiple of the medians (MoMs), which would simplify the implementation of screening tool [60•].

To our knowledge, no previous review has compared the prediction performance of ML to that of classical regression studies in pre-eclampsia prediction. We have captured previous studies that compared ML with classical regression studies in pre-eclampsia [131–134]. Similar to our review, a recent systematic review compared ML and classical regression studies in cardiovascular risk prediction and found that ML algorithms outperformed classical regression models [132, 135]. Other comparison reviews in hypertension [133] and acute kidney injury [33] found that ML algorithms had similar prediction performance to classical regression models, aligned to other clinical prediction models [32, 136]. However, a recent study reported that ML algorithms are a more powerful tool for prediction modelling than classical regression models in terms of higher flexibility and automatic data-dependent complexity optimisation [137]. Machine learning prediction can address challenges with rare events (class imbalance) prediction by oversampling the minority class and/or undersampling the majority class [138–140]. Classical regression models may be challenging to predict rare events, potentially yielding unstable prediction metrics values [141]. Consequently, advanced ML algorithms like random forest and boosting type algorithms might benefit from predicting rare events such as pre-eclampsia.

In this systematic review, we observed a lack of direct comparison between ML algorithms and classical regression models using harmonised data sources and evaluation metrics. Further research may focus on head-to-head comparisons using harmonised data sources and the same evaluation metrics, ideally measured on test rather than development data to minimise overfitting and consequently optimism. To gain a comprehensive understanding of true performance in other healthcare settings, it encourages research in low- and middle-income countries to apply these prediction models.

In terms of ML methods, similar to this review, some studies have shown that random forest and boosting-type algorithms (gradient boosting and extreme gradient boosting) achieve better prediction performance [33] compared with other ML approaches. Potentially, random forest and boosting-type algorithms are some of the most powerful algorithms, especially for structured and tabular data. Random forest is an ensemble learning algorithm that combines multiple decision trees based on bagging and random feature selection to make a prediction. As compared to other algorithms, random forests reduce overfitting, handle missing data, are robust to outliers, and can work out-of-the-box with less sensitive to hyperparameter selection [142]. Boosting-type algorithms such as gradient boosting and extreme gradient boosting are another class of ensemble learning starting with a weak algorithm (often decision tree) and sequentially boost its performance to create a stronger algorithm [143, 144]. As a result, boosting-type algorithms can handle imbalanced datasets, missing values, and allow for fine-grained control over hyperparameters for optimisation [145]. However, further algorithm development might be needed to differentiate the best algorithm for pre-eclampsia prediction; if this is confirmed, it would be advantageous (1) to externally validate the best-fit ML algorithm and (2) to facilitate clinical implementation in healthcare settings.

This study faces some limitations. Firstly, a high or unclear methodological risk of bias yet low concern for applicability was seen in both ML and classical regression studies. Some studies report insufficient sample sizes which might increase the risk of overfitting and can yield inaccurate and unstable predictions. Deployment strategies were seen in some classical regression models, but not in ML algorithms. ML algorithms lack interpretability, making it difficult to present equations and explicit mathematical relationships. Besides, the majority of the studies have not reported model’s calibration performance, which led to challenges in judging the accuracy of the risk estimates. Secondly, none of the ML studies reported external validation; hence, it remains unclear how well the models could perform among diverse population and settings. Therefore, further studies warranted for temporal and external validation. Furthermore, prediction performance can be influenced and underestimated by the treatment paradox, wherein high-risk women who would otherwise develop pre-eclampsia are treated with aspirin and do not develop the disease, effectively converting true-positives into false-positive results from predictive tests.

This review also has strengths. It was able to review the common prognostic factors in term of pre-eclampsia prediction, those were shown to consistent throughout studies to enhance practical of future prediction studies. Both prediction approaches were particularly compared against studies that used the same sample and similar prognostic factors, perhaps helpful in evaluating their performance in predicting the outcome of interest.

## Conclusion

This systematic review has explored prognostic factors and compared ML algorithms and classical regression models for pre-eclampsia prediction. Maternal demographic and clinical characteristics, MAP, UtA-PI, PAPP-A, and PIGF are the most used prognostic factors. Pre-eclampsia prediction performance appears better with ML algorithms, yet varies among ML approaches. Advanced ML algorithms such as random forest, gradient boosting, and extreme gradient boosting outperformed classical regression models in discrimination. To gain further insight into the performance of ML algorithms, research should focus on comparing ML algorithms to classical regression models using similar samples, evaluation metrics, comparing calibration, and conducting external validation of ML algorithms to provide insight into generalisability to other populations and settings. Ultimately, for optimal models, effective deployment and implementation strategies are needed.

### Supplementary Information

Below is the link to the electronic supplementary material.

Supplementary file1 (DOCX 141 KB)

## Data Availability

Available from the corresponding author upon reasonable request.

## References

[1] Lambert G, Brichant JF, Hartstein G, Bonhomme V, Dewandre PY (2014). Preeclampsia: an update. Acta Anaesthesiol Belg.

[2] Visintin C, Mugglestone MA, Almerie MQ, Nherera LM, James D, Walkinshaw S (2010). Management of hypertensive disorders during pregnancy: summary of NICE guidance. BMJ.

[3] Hypertension G (2020). Gestational hypertension and preeclampsia: ACOG Practice Bulletin Summary, Number 222. Obstet Gynecol.

[4] Karrar SA, Hong PL. Preeclampsia. InStatPearls [Internet] 2023 Feb 13. StatPearls Publishing.

[5] Poon LC, Shennan A, Hyett JA, Kapur A, Hadar E, Divakar H (2019). The International Federation of Gynecology and Obstetrics (FIGO) initiative on pre-eclampsia: a pragmatic guide for first-trimester screening and prevention. Int J Gynaecol Obstet Off organ Int Fed Gynaecol Obstet.

[6] Rolnik DL, Wright D, Poon LC, O’Gorman N, Syngelaki A, de Paco MC (2017). Aspirin versus placebo in pregnancies at high risk for preterm preeclampsia. N Engl J Med.

[7] Henderson JT, Whitlock EP, O’Connor E, Senger CA, Thompson JH, Rowland MG (2014). Low-dose aspirin for prevention of morbidity and mortality from preeclampsia: a systematic evidence review for the U.S. preventive services task force. Ann Intern Med.

[8] Van Doorn R, Mukhtarova N, Flyke IP, Lasarev M, Kim K, Hennekens CH (2021). Dose of aspirin to prevent preterm preeclampsia in women with moderate or high-risk factors: a systematic review and meta-analysis. PLoS ONE.

[9] Rolnik DL, Nicolaides KH, Poon LC (2022). Prevention of preeclampsia with aspirin. Am J Obstet Gynecol [Internet].

[10] Steyerberg EW. Clinical models prediction a practical approach to development, validation, and updating. 2019.

[11] Duckitt K, Harrington D (2005). Risk factors for pre-eclampsia at antenatal booking: systematic review of controlled studies. Br Med J.

[12] O’Brien TE, Ray JG, Chan W-S (2003). Maternal body mass index and the risk of preeclampsia: a systematic overview. Epidemiology.

[13] Bartsch E, Medcalf KE, Park AL, Ray JG, Al-Rubaie ZTA, Askie LM, et al. Clinical risk factors for pre-eclampsia determined in early pregnancy: systematic review and meta-analysis of large cohort studies. BMJ. 2016;353. 10.1136/bmj.i1753 PMC4837230 27094586

[14] Akolekar R, Syngelaki A, Poon L, Wright D, Nicolaides KH (2013). Competing risks model in early screening for preeclampsia by biophysical and biochemical markers. Fetal Diagn Ther.

[15] MacDonald TM, Walker SP, Hannan NJ, Tong S, Kaitu’u-Lino TJ (2022). Clinical tools and biomarkers to predict preeclampsia. eBioMedicine.

[16] Al-Rubaie ZTA, Askie LM, Ray JG, Hudson HM, Lord SJ (2016). The performance of risk prediction models for pre-eclampsia using routinely collected maternal characteristics and comparison with models that include specialised tests and with clinical guideline decision rules: a systematic review. BJOG An Int J Obstet Gynaecol.

[17] Riley RD, van der Windt D, Croft P, Moons KGM. Prognosis research in healthcare: concepts, methods, and impact. Oxford University Press; 2019.

[18] Scazzocchio E, Crovetto F, Triunfo S, Gratacós E, Figueras F (2017). Validation of a first-trimester screening model for pre-eclampsia in an unselected population. Ultrasound Obstet Gynecol.

[19] Tan MY, Syngelaki A, Poon LC, Rolnik DL, O’Gorman N, Delgado JL (2018). Screening for pre-eclampsia by maternal factors and biomarkers at 11–13 weeks’ gestation. Ultrasound Obstet Gynecol.

[20] Grobbee DE, Hoes AW. Clinical epidemiology: principles, methods, and applications for clinical research. Jones & Bartlett Publishers; 2014.

[21] Steyerberg EW, Moons KGM, van der Windt DA, Hayden JA, Perel P, Schroter S (2013). Prognosis Research Strategy (PROGRESS) 3: prognostic model research. PLoS Med.

[22] Zhang A, Xing L, Zou J, Wu JC (2022). Shifting machine learning for healthcare from development to deployment and from models to data. Nat Biomed Eng.

[23] Topol EJ (2019). High-performance medicine: the convergence of human and artificial intelligence. Nat Med.

[24] Yu KH, Beam AL, Kohane IS (2018). Artificial intelligence in healthcare. Nat Biomed Eng.

[25] Leo B (2001). Statistical modeling: the two cultures. Stat Sci.

[26] Halperin I (2020). Qlbs: Q-learner in the black-scholes (-merton) worlds. J Deriv.

[27] Jordan MI, Mitchell TM (2015). Machine learning: trends, perspectives, and prospects. Science..

[28] Dahan H, Cohen S, Rokach L, Maimon O. Proactive data mining using decision trees. Proactive Data Min with Decis Trees. Springer. 2014. p. 21–33.

[29] Goldstein BA, Navar AM, Carter RE (2017). Moving beyond regression techniques in cardiovascular risk prediction: applying machine learning to address analytic challenges. Eur Hear J..

[30] Luo W, Phung D, Tran T, Gupta S, Rana S, Karmakar C (2016). Guidelines for developing and reporting machine learning predictive models in biomedical research: a multidisciplinary view corresponding author.. J Med INTERNET Res.

[31] Beam AL, Kohane IS (2018). Big data and machine learning in health care. JAMA.

[32] Christodoulou E, Ma J, Collins GS, Steyerberg EW, Verbakel JY, Van Calster B (2019). REVIEW A systematic review shows no performance benefit of machine learning over logistic regression for clinical prediction models. J Clin Epidemiol..

[33] Song X, Liu X, Liu F, Wang C (2021). Comparison of machine learning and logistic regression models in predicting acute kidney injury: a systematic review and meta-analysis. Int J Med Inform [Internet]..

[34] De Kat AC, Hirst J, Woodward M, Kennedy S, Peters SA (2019). Prediction models for preeclampsia: a systematic review. Pregnancy Hypertens.

[35] Townsend R, Khalil A, Premakumar Y, Allotey J, Snell KIE, Chan C (2019). Prediction of pre-eclampsia: review of reviews. Ultrasound Obstet Gynecol.

[36] Antwi E, Amoakoh-Coleman M, Vieira DL, Madhavaram S, Koram KA, Grobbee DE (2020). Systematic review of prediction models for gestational hypertension and preeclampsia. PLoS One [Internet]..

[37] Page MJ, McKenzie JE, Bossuyt PM, Boutron I, Hoffmann TC, Mulrow CD, et al. The PRISMA 2020 statement: an updated guideline for reporting systematic reviews. BMJ [Internet]. 2021;372. Available from: http://www.prisma-statement.org/. 10.1136/bmj.n71 PMC8005924 33782057

[38] Debray TPA, Damen JAAG, Snell KIE, Ensor J, Hooft L, Reitsma JB, et al. A guide to systematic review and meta-analysis of prediction model performance. BMJ. 2017. 10.1136/bmj.i6460 28057641

[39] Dimitriadis E, Rolnik DL, Zhou W, Estrada-Gutierrez G, Koga K, Francisco RPV (2023). Pre-eclampsia. Nat Rev Dis Prim.

[40] Enticott J, Buck K, Shawyer F (2018). Finding, “hard to find” literature on hard to find groups: a novel technique to search grey literature on refugees and asylum seekers. Int J Methods Psychiatr Res.

[41] Geersing GJ, Bouwmeester W, Zuithoff P, Spijker R, Leeflang M, Moons K (2012). Search filters for finding prognostic and diagnostic prediction studies in medline to enhance systematic reviews. PLoS ONE.

[42] Covidence systematic review software, Veritas Health Innovation, Melbourne, Australia. Available at.

[43] Wolff RF, Moons KGM, Riley RD, Whiting PF, Westwood M, Collins GS (2019). PROBAST: a tool to assess the risk of bias and applicability of prediction model studies. Ann Intern Med.

[44] Moons KGM, de Groot JAH, Bouwmeester W, Vergouwe Y, Mallett S, Altman DG (2014). Critical appraisal and data extraction for systematic reviews of prediction modelling studies: the CHARMS checklist. PLoS Med.

[45] Hosmer Jr DW, Lemeshow S, Sturdivant RX. Applied logistic regression. John Wiley & Sons. 2013.

[46] Steyerberg EW. Clinical prediction models: a practical approach to development, validation and updating. Biometrics. 2010. 10.1111/j.1541-0420.2010.01431.x.

[47] Van Calster B, McLernon DJ, Van Smeden M, Wynants L, Steyerberg EW, Bossuyt P (2019). Calibration: the Achilles heel of predictive analytics. BMC Med.

[48] Liu M, Yang X, Chen G, Ding Y, Shi M, Sun L (2022). Development of a prediction model on preeclampsia using machine learning-based method: a retrospective cohort study in China. Front Physiol..

[49] Li YX, Shen XP, Yang C, Cao ZZ, Du R, Yu MD (2021). Novel electronic health records applied for prediction of pre-eclampsia machine-learning algorithms. Pregnancy Hypertens.

[50] Zhang X, Chen Y, Salerno S, Li Y, Zhou L, Zeng X (2022). Prediction of severe preeclampsia in machine learning. Med Nov Technol Devices.

[51] Marić I, Tsur A, Aghaeepour N, Montanari A, Stevenson DK, Shaw GM (2020). Early prediction of preeclampsia via machine learning. Am J Obstet Gynecol MFM.

[52] Bennett R, Mulla ZD, Parikh P, Hauspurg A, Razzaghi T (2022). An imbalance-aware deep neural network for early prediction of preeclampsia [Internet]. PLoS One.

[53] Marin I, Pavaloiu BI, Marian CV, Racovita V, Goga N. Early detection of preeclampsia based on a machine learning approach. 2019 7th E-Health Bioeng Conf EHB 2019. 2019;21–4.

[54] Sufriyana H, Wu YW, Su ECY. Artificial intelligence-assisted prediction of preeclampsia: development and external validation of a nationwide health insurance dataset of the BPJS Kesehatan in Indonesia. EBioMedicine. 2020a;54. 10.1016/j.ebiom.2020.102710 PMC7152721 32283530

[55] Sufriyana H, Wu YW, Su ECY (2020). Prediction of preeclampsia and intrauterine growth restriction: development of machine learning models on a prospective cohort. JMIR Med Inform.

[56] Gómez-Jemes  L, Oprescu AM, Chimenea-Toscano Á, García-Díaz L, Romero-Ternero MDC (2022). Machine learning to predict pre-eclampsia and intrauterine growth restriction in pregnant women. Electronics.

[57] Jhee JH, Lee S, Park Y, Lee SE, Kim YA, Kang S-W (2019). Prediction model development of late-onset preeclampsia using machine learning-based methods. PLoS ONE.

[58] Sandström A, Snowden JM, Höijer J, Bottai M, Wikström AK (2019). Clinical risk assessment in early pregnancy for preeclampsia in nulliparous women: a population based cohort study. PLoS ONE.

[59] Melinte-Popescu AS, Vasilache IA, Socolov D, Melinte-Popescu M (2023). Predictive performance of machine learning-based methods for the prediction of preeclampsia—a prospective study. J Clin Med.

[60] Ansbacher-Feldman Z, Syngelaki A, Meiri H, Cirkin R, Nicolaides KH, Louzoun Y (2022). Machine-learning-based prediction of pre-eclampsia using first-trimester maternal characteristics and biomarkers. Ultrasound Obstet Gynecol..

[61] Chen X, Yuan L, Ji Z, Bian X, Hua S (2022). Development and validation of the prediction models for preeclampsia: a retrospective, single-center, case-control study. Ann Transl Med.

[62] Wanriko S, Hnoohom N, Wongpatikaseree K, Jitpattanakul A, Musigavong O. Risk assessment of pregnancy-induced hypertension using a machine learning approach. 2021 Jt 6th Int Conf Digit Arts, Media Technol with 4th ECTI North Sect Conf Electr Electron Comput Telecommun Eng ECTI DAMT NCON 2021. 2021;233–7.

[63] Manoochehri Z, Manoochehri S, Soltani F, Tapak L, Sadeghifar M (2021). Predicting preeclampsia and related risk factors using data mining approaches: a cross-sectional study. Int J Reprod Biomed.

[64] Bunyapipat P, Pruksanusak N, Suwanrath C, Geater A (2023). Combined maternal risk factors and the Quadruple test to predict late-onset preeclampsia in pregnant Thai women. BMC Pregnancy Childbirth.

[65] Suksai M, Geater A, Phumsiripaiboon P, Suntharasaj T (2022). A new risk score model to predict preeclampsia using maternal factors and mean arterial pressure in early pregnancy. J Obstet Gynaecol (Lahore).

[66] Tarca AL, Taran A, Romero R, Jung E, Paredes C, Bhatti G (2022). Prediction of preeclampsia throughout gestation with maternal characteristics and biophysical and biochemical markers: a longitudinal study. Am J Obstet Gynecol [Internet]..

[67] Tang Z, Ji Y, Zhou S, Su T, Yuan Z, Han N (2022). Development and validation of multi-stage prediction models for pre-eclampsia: a retrospective cohort study on Chinese women. Front Public Heal.

[68] Yue CY, Gao JP, Zhang CY, Ni YH, Ying CM (2021). Development and validation of a nomogram for the early prediction of preeclampsia in pregnant Chinese women. Hypertens Res [Internet].

[69] Kim YR, Jung I, Park G, Chang SW, Cho HY (2021). First-trimester screening for early preeclampsia risk using maternal characteristics and estimated placental volume. J Matern Neonatal Med.

[70] Wang W, Wang Y, Yuan T, Zhang H, Li C, Li X (2020). Nomogram-based prediction of pre-eclampsia in the first trimester of gestation. Pregnancy Hypertens.

[71] Al-Rubaie ZTA, Hudson HM, Jenkins G, Mahmoud I, Ray JG, Askie LM (2020). Prediction of pre-eclampsia in nulliparous women using routinely collected maternal characteristics: a model development and validation study. BMC Pregnancy Childbirth.

[72] Sovio U, Smith GCS (2019). Evaluation of a simple risk score to predict preterm pre-eclampsia using maternal characteristics: a prospective cohort study. BJOG An Int J Obstet Gynaecol.

[73] Boutin A, Demers S, Gasse C, Giguère Y, Tétu A, Laforest G (2019). First-trimester placental growth factor for the prediction of preeclampsia in Nulliparous women: the great obstetrical syndromes cohort study. Fetal Diagn Ther.

[74] Boutin A, Gasse C, Demers S, Giguère Y, Tétu A, Bujold E (2018). Maternal characteristics for the prediction of preeclampsia in nulliparous women: the great obstetrical syndromes (GOS) study. J Obstet Gynaecol Canada.

[75] Cheng YKY, Leung TY, Law LW, Ting YH, Law KM, Sahota DS (2018). First trimester screening for pre-eclampsia in Chinese pregnancies: case–control study. BJOG An Int J Obstet Gynaecol.

[76] Praciano De Souza PC, Gurgel Alves JA, Maia BE, Moura SH, Júnior AE, Martins WP, Silva Costa FD (2018). Second trimester screening of preeclampsia using maternal characteristics and uterine and ophthalmic artery Doppler. Ultraschall der Medizin.

[77] Asiltas B, Surmen-Gur E, Uncu G (2018). Prediction of first-trimester preeclampsia: relevance of the oxidative stress marker MDA in a combination model with PP-13, PAPP-A and beta-HCG. Pathophysiology [Internet]..

[78] Rocha RS, Alves JAG, Holanda Moura SBME, Araujo E, Peixoto AB, Santana EFM (2017). Simple approach based on maternal characteristics and mean arterial pressure for the prediction of preeclampsia in the first trimester of pregnancy. J Perinat Med.

[79] Luo Q, Han X (2017). Second-trimester maternal serum markers in the prediction of preeclampsia. J Perinat Med.

[80] Agarwal R, Chaudhary S, Kar R, Radhakrishnan G, Tandon A (2017). Prediction of preeclampsia in primigravida in late first trimester using serum placental growth factor alone and by combination model. J Obstet Gynaecol (Lahore).

[81] Guy GP, Ling HZ, Garcia P, Poon LC, Nicolaides KH (2017). Maternal cardiac function at 35–37 weeks’ gestation: prediction of pre-eclampsia and gestational hypertension. Ultrasound Obstet Gynecol.

[82] Gabbay-Benziv R, Oliveira N, Baschat AA (2016). Optimal first trimester preeclampsia prediction: a comparison of multimarker algorithm, risk profiles and their sequential application. Prenat Diagn.

[83] Kumar M, Sharma K, Singh S, Ravi V, Singh K (2016). Role of maternal factors, PAPP-A, and Doppler in screening for early- and late-onset pregnancy hypertension in Asian population. Hypertens Pregnancy.

[84] Giguere Y, Masse J, Theriault S, Bujold E, Lafond J, Rousseau F (2015). Screening for pre-eclampsia early in pregnancy: performance of a multivariable model combining clinical characteristics and biochemical markers. BJOG An Int J Obstet Gynaecol.

[85] Wright D, Syngelaki A, Akolekar R, Poon LC, Nicolaides KH (2015). Competing risks model in screening for preeclampsia by maternal characteristics and medical history. Am J Obstet Gynecol [Internet]..

[86] Moon M, Odibo A (2015). First-trimester screening for preeclampsia: impact of maternal parity on modeling and screening effectiveness. J Matern Neonatal Med.

[87] Baschat AA, Magder LS, Doyle LE, Atlas RO, Jenkins CB, Blitzer MG (2014). Prediction of preeclampsia utilizing the first trimester screening examination. Am J Obstet Gynecol [Internet]..

[88] Kenny LC, Black MA, Poston L, Taylor R, Myers JE, Baker PN (2014). Early pregnancy prediction of preeclampsia in nulliparous women, combining clinical risk and biomarkers: the Screening for Pregnancy Endpoints (SCOPE) international cohort study. Hypertension.

[89] Goetzinger KR, Tuuli MG, Cahill AG, Macones GA, Odibo AO (2014). Development and validation of a risk factor scoring system for first-trimester prediction of preeclampsia. Am J Perinatol.

[90] Gurgel Alves JA, Praciano de Sousa PC, Maia BE, Moura HS, Kane SC, Silva Costa FD (2014). First‐trimester maternal ophthalmic artery Doppler analysis for prediction of pre‐eclampsia. Ultrasound Obstet Gynecol.

[91] Teixeira C, Tejera E, Martins H, Pereira AT, Costa-Pereira A, Rebelo I (2014). First trimester aneuploidy screening program for preeclampsia prediction in a Portuguese obstetric population. Obstet Gynecol Int.

[92] Skråstad RB, Hov GG, Blaas HGK, Romundstad PR, Salvesen KA (2014). A prospective study of screening for hypertensive disorders of pregnancy at 11–13 weeks in a Scandinavian population. Acta Obstet Gynecol Scand.

[93] Direkvand-Moghadam A, Khosravi A, Sayehmiri K (2013). Predictive factors for preeclampsia in pregnant women: a Receiver Operation Character approach. Arch Med Sci.

[94] North RA, McCowan LME, Dekker GA, Poston L, Chan EHY, Stewart AW, et al. Clinical risk prediction for pre-eclampsia in nulliparous women: development of model in international prospective cohort. Bmj. 2011;342. 10.1136/bmj.d1875 PMC3072235 21474517

[95] Odibo AO, Zhong Y, Goetzinger KR, Odibo L, Bick JL, Bower CR (2011). First-trimester placental protein 13, PAPP-A, uterine artery Doppler and maternal characteristics in the prediction of pre-eclampsia. Placenta [Internet]..

[96] Yu J, Shixia CZ, Wu Y, Duan T (2011). Inhibin A, activin A, placental growth factor and uterine artery Doppler pulsatility index in the prediction of pre-eclampsia. Ultrasound Obstet Gynecol.

[97] Goetzinger KR, Singla A, Gerkowicz S, Dicke JM, Gray DL, Odibo AO (2010). Predicting the risk of pre-eclampsia between 11 and 13 weeks’ gestation by combining maternal characteristics and serum analytes. PAPP-A and free β-hCG Prenat Diagn.

[98] Thilaganathan B, Wormald B, Zanardini C, Sheldon J, Ralph E, Papageorghiou AT (2010). Early-pregnancy multiple serum markers and second-trimester uterine artery doppler in predicting preeclampsia. Obstet Gynecol.

[99] Poon LCY, Kametas NA, Pandeva I, Valencia C, Nicolaides KH (2008). Mean arterial pressure at 11+0 to 13+6 weeks in the prediction of preeclampsia. Hypertension.

[100] Deis S, Rouzier R, Kayem G, Masson C, Haddad B (2008). Development of a nomogram to predict occurrence of preeclampsia. Eur J Obstet Gynecol Reprod Biol.

[101] De Paco C, Kametas N, Rencoret G, Strobl I, Nicolaides KH (2008). Maternal cardiac output between 11 and 13 weeks of gestation in the prediction of preeclampsia and small for gestational age. Obstet Gynecol.

[102] Pilalis A, Souka AP, Antsaklis P, Daskalakis G, Papantoniou N, Mesogitis S (2007). Screening for pre-eclampsia and fetal growth restriction by uterine artery Doppler and PAPP-A at 11–14 weeks’ gestation. Ultrasound Obstet Gynecol Off J Int Soc Ultrasound Obstet Gynecol.

[103] Yu LL, Fassett JD, MacDonald BS, Butler TA, Ramsey DM, Key-Schwartz RJ (2005). Development of SRMs 295x and 296x, respirable crystalline silica on filter. J ASTM Int.

[104] Papageorghiou AT, Yu CKH, Erasmus IE, Cuckle HS, Nicolaides KH (2005). Assessment of risk for the development of pre-eclampsia by maternal characteristics and uterine artery Doppler. BJOG An Int J Obstet Gynaecol.

[105] Harrington K, Carpenter RG, Goldfrad C, Campbell S (1997). Transvaginal doppler ultrasound of the uteroplacental circulation in the early prediction of pre-eclampsia and intrauterine growth retardation. BJOG An Int J Obstet Gynaecol.

[106] Serra B, Mendoza M, Scazzocchio E, Meler E, Nolla M, Sabrià E (2020). A new model for screening for early-onset preeclampsia. Am J Obstet Gynecol [Internet]..

[107] Crovetto F, Figueras F, Triunfo S, Crispi F, Rodriguez-sureda V, Dominguez C (2015). First trimester screening for early and late preeclampsia based on maternal characteristics, biophysical parameters, and angiogenic factors. Prenat Diagn.

[108] Yliniemi A, Makikallio K, Korpimaki T, Kouru H, Marttala J, Ryynanen M. Combination of PAPPA, fhCGβ, AFP, PIGF, sTNFR1, and maternal characteristics in prediction of early-onset preeclampsia. Clin Med Insights Reprod Heal. 2015;9:CMRH.S21865. 10.4137/CMRH.S21865 PMC4469033 26106266

[109] Crovetto F, Figueras F, Triunfo S, Crispi F, Rodriguez-Sureda V, Peguero A (2014). Added value of angiogenic factors for the prediction of early and late preeclampsia in the first trimester of pregnancy. Fetal Diagn Ther.

[110] Scazzocchio E, Figueras F, Crispi F, Meler E, Masoller N, Mula R (2013). Performance of a first-trimester screening of preeclampsia in a routine care low-risk setting. Am J Obstet Gynecol [Internet].

[111] Parra-Cordero M, Rodrigo R, Barja P, Bosco C, Rencoret G, Sepúlveda-Martinez A (2013). Prediction of early and late pre-eclampsia from maternal characteristics, uterine artery Doppler and markers of vasculogenesis during first trimester of pregnancy. Ultrasound Obstet Gynecol.

[112] Kuc S, Koster MPH, Franx A, Schielen PCJI, Visser GHA (2013). Maternal characteristics, mean arterial pressure and serum markers in early prediction of preeclampsia. PLoS ONE.

[113] Caradeux J, Serra R, Nien J-K, Pérez-Sepulveda A, Schepeler M, Guerra F (2013). First trimester prediction of early onset preeclampsia using demographic, clinical, and sonographic data: a cohort study. Prenat Diagn.

[114] Di Lorenzo G, Ceccarello M, Cecotti V, Ronfani L, Monasta L, Brumatti LV (2012). First trimester maternal serum PIGF, free β-hCG, PAPP-A, PP-13, uterine artery Doppler and maternal history for the prediction of preeclampsia. Placenta [Internet].

[115] Akolekar R, Syngelaki A, Sarquis R, Zvanca M, Nicolaides KH (2011). Prediction of early, intermediate and late pre-eclampsia from maternal factors, biophysical and biochemical markers at 11–13 weeks. Prenat Diagn.

[116] Kuijk SMJV, Nijdam ME, Janssen KJM, Sep SJS, Peeters LL, Delahaije DHJ (2011). A model for preconceptional prediction of recurrent early-onset preeclampsia: derivation and internal validation. Reprod Sci.

[117] Poon LCY, Staboulidou I, Maiz N, Plasencia W, Nicolaides KH (2009). Hypertensive disorders in pregnancy: screening by uterine artery Doppler at 11–13 weeks. Ultrasound Obstet Gynecol.

[118] Poon LCY, Maiz N, Valencia C, Plasencia W, Nicolaides KH (2009). First-trimester maternal serum pregnancy-associated plasma protein-A and pre-eclampsia. Ultrasound Obstet Gynecol.

[119] Poon LCY, Kametas NA, Maiz N, Akolekar R, Nicolaides KH (2009). First-trimester prediction of hypertensive disorders in pregnancy. Hypertension.

[120] Akolekar R, Zaragoza E, Poon LCY, Pepes S, Nicolaides KH (2008). Maternal serum placental growth factor at 11 + 0 to 13 + 6 weeks of gestation in the prediction of pre-eclampsia. Ultrasound Obstet Gynecol.

[121] Plasencia W, Maiz N, Poon L, Yu C, Nicolaides KH (2008). Uterine artery Doppler at 11 + 0 to 13 + 6 weeks and 21 + 0 to 24 + 6 weeks in the prediction of pre-eclampsia. Ultrasound Obstet Gynecol.

[122] Onwudiwe N, Yu CKH, Poon LCY, Spiliopoulos I, Nicolaides KH (2008). Prediction of pre-eclampsia by a combination of maternal history, uterine artery Doppler and mean arterial pressure. Ultrasound Obstet Gynecol Off J Int Soc Ultrasound Obstet Gynecol.

[123] Youssef A, Righetti F, Morano D, Rizzo N, Farina A (2011). Uterine artery Doppler and biochemical markers (PAPP-A, PlGF, sFlt-1, P-selectin, NGAL) at 11+ 0 to 13+ 6 weeks in the prediction of late (> 34 weeks) pre-eclampsia. Prenat Diagn.

[124] Sandström A, Snowden  JM, Bottai M, Stephansson O, Wikström AK, Li YX (2022). An imbalance-aware deep neural network for early prediction of preeclampsia. PLoS One [Internet].

[125] Pihl K, Sørensen S, Stener JF (2020). Prediction of preeclampsia in nulliparous women according to first trimester maternal factors and serum markers. Fetal Diagn Ther.

[126] Sepúlveda-Martínez A, Rencoret G, Silva MC, Ahumada P, Pedraza D, Muñoz H (2019). First trimester screening for preterm and term pre-eclampsia by maternal characteristics and biophysical markers in a low-risk population. J Obstet Gynaecol Res.

[127] Myers JE, Kenny LC, McCowan LME, Chan EHY, Dekker GA, Poston L (2013). Angiogenic factors combined with clinical risk factors to predict preterm pre-eclampsia in nulliparous women: a predictive test accuracy study. BJOG An Int J Obstet Gynaecol.

[128] Wright D, Akolekar R, Syngelaki A, Poon LCY, Nicolaides KH (2012). A competing risks model in early screening for preeclampsia. Fetal Diagn Ther.

[129] Sarker IH (2021). Machine learning: algorithms, real-world applications and research directions. SN Comput Sci [Internet]..

[130] Habehh H, Gohel S (2021). Machine learning in healthcare. Curr Genomics.

[131] Sun Z, Dong W, Shi H, Ma H, Cheng L, Huang Z (2022). Comparing machine learning models and statistical models for predicting heart failure events: a systematic review and meta-analysis. Front Cardiovasc Med.

[132] Liu W, Laranjo L, Klimis H, Chiang J, Yue J, Marschner S (2023). Machine-learning versus traditional approaches for atherosclerotic cardiovascular risk prognostication in primary prevention cohorts: a systematic review and meta-analysis. Eur Hear J - Qual Care Clin Outcomes.

[133] Chowdhury MZI, Naeem I, Quan H, Leung AA, Sikdar KC, OBeirne M, et al. Prediction of hypertension using traditional regression and machine learning models: a systematic review and meta-analysis. PLoS One [Internet]. 2022;17. Available from: 10.1371/journal.pone.0266334 10.1371/journal.pone.0266334 PMC8989291 35390039

[134] Gravesteijn BY, Nieboer D, Ercole A, Lingsma HF, Nelson D, van Calster B (2020). Machine learning algorithms performed no better than regression models for prognostication in traumatic brain injury. J Clin Epidemiol.

[135] Talwar A, Lopez-olivo MA, Huang Y, Ying L, Aparasu RR (2023). Exploratory research in clinical and social pharmacy performance of advanced machine learning algorithms overlogistic regression in predicting hospital readmissions: a meta-analysis. Explor Res Clin Soc Pharm [Internet].

[136] Clift AK, Dodwell D, Lord S, Petrou S, Brady M, Collins GS (2023). Development and internal-external validation of statistical and machine learning models for breast cancer prognostication: cohort study. BMJ.

[137] Pichler M, Hartig F (2023). Machine learning and deep learning—a review for ecologists. Methods Ecol Evol.

[138] Blagus R, Lusa L (2017). Gradient boosting for high-dimensional prediction of rare events. Comput Stat Data Anal [Internet].

[139] Lunardon N, Menardi G, Torelli N. R package’ROSE’: random over-sampling examples. 2013.

[140] Blagus R, Lusa L (2015). Joint use of over-and under-sampling techniques and cross-validation for the development and assessment of prediction models. BMC Bioinformatics.

[141] Feng C, Li L, Xu C (2023). Advancements in predicting and modeling rare event outcomes for enhanced decision-making. BMC Med Res Methodol [Internet]..

[142] Breiman L (2001). Random forests. Mach Learn.

[143] Friedman JH. Greedy function approximation: a gradient boosting machine. Ann Stat. 2001;1189–232.

[144] Chen T, Guestrin C. Xgboost: a scalable tree boosting system. Proc 22nd acm sigkdd Int Conf Knowl Discov data Min. 2016. p. 785–94.

[145] Bentéjac C, Csörgő A, Martínez-Muñoz G (2021). A comparative analysis of gradient boosting algorithms. Artif Intell Rev.

