# Supplementary material for: Machine Learning Algorithms Versus Classical Regression Models in Pre-Eclampsia Prediction: A Systematic Review
Source: Curr Hypertens Rep. 2024 May 28;26(7):309–23. doi: 10.1007/s11906-024-01297-1 (PMC11199280; doi:10.1007/s11906-024-01297-1)
Supplement: Supplementary file 1 — Supplementary file1 (DOCX 141 KB) [file 11906_2024_1297_MOESM1_ESM.docx]

Supplementary Table 1: Detail characteristics of machine learning (ML) prediction studies

| **Study** | **Timing of variables measurement** | **Model development algorithm approaches** | **Algorithm calibration** | **Model validation:**  **Internal or External** | **Deployment strategies** |
| --- | --- | --- | --- | --- | --- |
| Melinte-Popescu *et al.*, 2023 | First trimester | DT, NB, SVM, RF* | NR | NR | Not reported |
| Liu et al., 2022 | 11 – 13^+6^ weeks | LR, RF*, DT, SVM, DNN | Slope 0.92 | Internal, 10-fold cross-validation | Not reported |
| Zhang et al., 2022 | 10 – 15 weeks | DT, RF, LightGBM* | NR | Internal, split sample | Not reported |
| Gómez-Jemes et al., 2022 | 24 to 38 weeks | DT* | NR | Internal, split sample | Not reported |
| Bennett et al., 2022 | NR | DNN* | NR | Internal, split sample | Not reported |
| Ansbacher-Feldman et al., 2022 | 11 – 13 weeks | nnet* | NR | Internal, split sample | Not reported |
| Chen *et al.*, 2022 | - | LR, DT, RF* | NR | Internal, split sample | Not reported |
| Li et al., 2021 | 10 – 13^+6^ weeks | LR, RF, SVM, XGBoost* | Brier score = 0.033 | Internal, 5-fold cross-validation and temporal validation | Not reported |
| Wanriko et al., 2021 | First trimester | LR, KNN, DT, RF*, MLN, SVM, NB | NR | Internal, split sample | Not reported |
| Manoochehri et al., 2021 | First trimester | LR, KNN, DT, LDA, RF, SVM* | NR | Internal, split sample | Not reported |
| Marić et al., 2020 | < 16 weeks | Elastic net, GB*, LR | NR | Internal, split sample | Not reported |
| Sufriyana, Wu and Su, 2020a | 11 – 13^+6^ weeks | RF, ANN, SVM, DT | Slope = 1.54 | Internal, split sample | Not reported |
| Sufriyana, Wu and Su, 2020b | - | CVR, RF*, NB | Well calibrated | Internal, 10-fold cross-validation | Not reported |
| Marin et al., 2019 | - | Viterbi ML algorithm* | NR | NR | Not reported |
| Jhee et al., 2019 | - | DT, NB, SVM, RF, GB* | NR | Internal, split sample | Not reported |
| Sandström *et al.*, 2019 | 11 – 13^+6^ weeks | LR*, RF | NR | Internal, 10-fold cross-validation | Not reported |

NB: * = the better performing ML algorithm, LR = Logistic Regression, DT = Decision Tree, RF = Random Forest, NB = Naïve Bayes, nnet = Neural networks, DNN = Deep Neural Network, SVM = Support Vector Machine, XGBoost = Extreme Gradient Boosting, GB = Gradient Boosting, CVR = Classification via Regression, KNN = K-Nearest neighbour, SVM = support vector machines, MLN = multilayer perceptron neural network, NR = Not Reported

Supplementary Table 2: Further characteristics of the included prognostic model studies.

| **Author, year** | **Timing of variable measurement** | **Linear predictor equations** | **Model approach** | **Deployment strategies** | **Model calibration** |
| --- | --- | --- | --- | --- | --- |
| **Classical-based prognostic models for any-onset pre-eclampsia** | | | | | |
| Suksai et al., 2022 | NR | Score = 3*maternal age ≥ 30 years +2*BMI ≥ 25 kg/m^2^ + 9*multifetal pregnancy + 9* Prior PE + 6*prior adverse pregnancy outcome +5*pregnancy interval > 10 years + 5*nulliparous + 10 *renal disease + 6*Chronic hypertension + systemic lupus erythematosus or antiphospholipid syndrome + 2*diabetes mellitus 5*mean arterial pressure ≥ 95mmHg | LR | Score chart rule | NR |
| Tarca et al., 2022 | 6 – 22 weeks | - | LR | RF | NR |
| Tang et al., 2022 | 14–18 weeks | Y = (−15.10) + (0.0890∗BMI) + (−0.888 if Multiparous) + (0.0479∗Maternal Age) + (0.407∗ if Pre–gestational diabetes mellitus) + (0.319∗if Family history of hypertension) + (0.0233∗ MAP measured at 5–10 weeks) + (0.0503∗MAP measured at 11–13 weeks) + (0.00609∗uric acid test at 11–13 weeks). | LR | RF | NR |
| Yue et al., 2021 | 14 – 24 weeks | Based on the nomogram | LR | Nomogram | Calibrated |
| Kim et al., 2021 | 11 – 13^+6^ weeks | - | LR | NR | NR |
| Wang et al., 2020 | First trimester | Based on the nomogram | LR | Nomogram | Calibrated |
| Al-Rubaie et al., 2020 | NR | LP = − 7.786 + 0.052 * maternal age in years from age 27 years + 0.078 * BMI in kg/m2 from 26.3 kg/m2 + 0.525 if Australian/New Zealand born English speaker + 1.318 if multiple pregnancy + 1.740 if family history of pre-eclampsia + 1.512 if autoimmune disease + 1.545 if chronic hypertension + 1.494 if chronic renal disease | LR | RF | Calibrated (p-value = 0.44) |
| Sovio and Smith, 2019 | 11 – 13^+6^ weeks | Score = if age of mother (<36 = 0, 36 = 1, 37 = 2, 38 = 3, 39 = 4, 40 = 5, 41 = 6, 42 = 7, 43 = 8, 44 = 9, ≥ 45 = 10) + if height of mother (<148 = 19, 148–149 = 18, 150–151 = 17, 152–153 = 16, 154–155 = 15, 156–157 = 14, 158–159 = 13, 160–161 = 12, 162–163 = 11, 164–165 = 10, 166–167 = 9, 168–169 = 8, 170–171 = 7, 172–173 = 6, 174–175 = 5, 176–177 = 4, 178–179 = 3, 180–181 = 2, 182–183 = 1, ≥184 = 0) + ethnicity (Afro-Caribbean = 13, South Asian = 5, Other = 0) + 35*Chronic hypertension + 15 Systemic lupus erythematosus or antiphospholipid syndrome + 8* Conception by IVF + weight if (<45 = 0, 45–47 = 1, 48–50 = 2, 51–53 = 3, 54–56 = 4, 57–59 = 5, 60–62 = 6, 63–65 = 7, 66–68 = 8, 69–71 = 9, 72–74 = 10, 75–77 = 11, 78–80 = 12, 81–83 = 13, 84–86 = 14, 87–89 = 15, 90–92 = 16, 93–95 = 17, 96–98 = 18, 99–101 = 19, 102–104 = 20, 105–107 = 21, 108–110 = 22, 111–113 = 23, 114–116 = 24, 117–119 = 25, ≥120 = 26) + 8* (Family history of pre-eclampsia) + 16*Diabetes mellitus type 1 or 2 | LR | Score chart rule | NR |
| Boutin et al., 2019 | First trimester | - | LR | NR | NR |
| Boutin et al., 2018 | 11 – 13^+6^ weeks | - | LR | NR | NR |
| Cheng et al., 2018 | NR | LP = -10.0166 + 0.1703*age - 1.1162(if parous with no PE) | LR | RF | NR |
| [Da Silva Costa](https://research.monash.edu/en/persons/fabricio-da-silva-costa) F et al., 2018 | Second trimester | - | LR | NR | NR |
| Asiltas et al., 2018 | 11 – 13^+6^ weeks | - | LR | NR | NR |
| Rocha et al., 2017 | 11 – 13^+6^ weeks | LP = –0.426 + 0.004 ×MAP + 0.129 (family history of PE) −0.059 (no prior PE) + 0.006 × BMI. | LR | RF | NR |
| Luo and Han, 2017 | 24 – 28 weeks | - | LR | NR | NR |
| Agarwal et al., 2017 | 11 – 13^+6^ weeks | - | LR | NR | NR |
| Guy et al., 2017 | 19 – 24 weeks | - | LR | NR | NR |
| Gabbay‐Benziv., 2016 | 9 – 13^+6^ weeks | - | LR | NR | NR |
| Kumar et al., 2016 | 11 – 13^+6^ weeks | - | LR | NR | NR |
| Giguère et al., 2015 | NR | - | LR | NR | NR |
| Wright et al., 2015 | 11 – 13^+6^ weeks | - | CRM | NR | NR |
| Moon and Odibo, 2015 | 11 – 13^+6^ weeks | - | LR | NR | NR |
| Baschat et al., 2014 | First trimester | LP = -8.72 + 0.157 (If nulliparous) + 0.341(if history of hypertension) + 0.635 (if prior PE) + 0.064 (MAP) - 0.186 (PAPP-A) | LR | RF | NR |
| Kenny et al., 2014 | 14 – 16 weeks | - | LR | NR | NR |
| Goetzinger et al., 2014 | NR | Score = 4* if chronic HTN + 3 * prior PE + 2*pre-gestational diabetes + BMI ≥ 30kg/m^2^ + 1*PAPP-A MoM < 10th percentile + 1* Bilateral uterine artery notching | LR | Score chart rule | Calibrated (P=0.25) |
| Gurgel Alves et al., 2014 | 11 – 13^+6^ weeks | - | LR | NR | NR |
| Teixeira et al., 2014 | 9 – 13^+6^ weeks | LP = -5.723 + 0.87* Chronic Hypertension + 1.428 * Diabetes mellitus -0.787 * parity + 3.952* Prior PE + 0.039 * maternal age + 6.159*maternal weight + 0.027 * crown rump length (CRL)-0.483* nuchal translucency thickness (NT)+ 0.766* Free 𝛽-hCG (MoM, log) | LR | RF | NR |
| Skråstad et al., 2014 | 11 – 13^+6^ weeks | For pre-eclampsia, LP = -11.6+MAP*0.07+UtAPI*1.3+Age*1.4  for sever pre-eclampsia, LP = -15.3+MAP*0.1+UtAPI*2.3+PlGF MoM* 4.1+PAPP-AMoM*1.1 | LR | RF | NR |
| Direkvand et al., 2013 | NR | LP = 0.74 –1.016 prior infertility + 0.72 chronic hypertension + 1.69 prior preeclampsia | LR | RF | NR |
| North et al., 2011 | 14 – 21 weeks | LP = 6.8855 − 0.0393×age + 0.0659×MAP + 0.0483×BMI + 0.6861×family history of preeclampsia + 0.6232 × family history of coronary heart disease − 0.3881×participant’s birth weight (kg) + 0.7129×vaginal bleeding ≥5 days − 0.8033×one miscarriage ≤10 weeks, same partner − 0.9070×≥12 months to conceive − 0.3733×high fruit intake at 15 weeks − 0.508×alcohol consumption in first trimester − 0.063×No of cigarettes/day at 15 weeks. | LR | RF | Not calibrated |
| Odibo et al., 2011 | First trimester | LP = 1.308 - 0.574 * PP13 MoM - 0.502* PAPP-A MoM -0.643 * Mean uterine artery PI + 0.799 if pre-gestational diabetic + 0.664 if race is black + 1.340 if history of chronic Hypertension | LR | RF | NR |
| Yu et al., 2011 | 12 – 16 weeks | - | LR | NR | NR |
| Goetzinger et al., 2010 | 11 – 13^+6^ weeks | LP = −3.25 + 0.51 × PAPP-A (1 if <10^th^ percentile, 0 if >10th percentile) + 0.93 × BMI (1 if >25, 0 if ≤25) + 0.94 × cHTN (1 if cHTN present, 0 if absent) + 0.97 × diabetes mellitus (DM) (1 if DM present, 0 if absent) + 0.61 × African American race (1 if African American, 0 if other race) | LR | RF | NR |
| Thilaganathan et al., 2010 | 11 – 13^+6^ weeks | - | LR | NR | NR |
| Poon et al., 2008 | 11 – 13^+6^ weeks | LP = -6.311 + (1.299 if Afro-Caribbean or 0 if other  ethnic origin) + 0.092 (BMI in kg/m2) + (0.855 if woman’s  Mother had PE) + (-1.481 if parous without previous PE, 0.933 if parous with previous PE, or 0 if nulliparous). | LR | RF | NR |
| Deis et al., 2008 | First trimester | Based on the nomogram | LR | Nomogram | Calibrated |
| De Paco et al., 2008 | 11 – 13^+6^ weeks | LP = -5.834 + 0.026* weight + 1.35*Afro-Caribbean + 1.392*Mixed ethnicity – 1.454 *parous with no prior PE + 1.071 *Parous with prior PE + family history of PE + 3.846* MoM cardiac output | LR | RF | NR |
| Pilalis et al., 2007 | 11 – 13^+6^ weeks | - | LR | NR | NR |
| Yu et al., 2005 | 11 – 13^+6^ weeks | LP = 1.8552 + (5.9228*Mean uterine artery pulsatility index ^^- 2^) + (-14.4474*Mean uterine artery pulsatility index ^^-1^) + (-0.5478 *smoker) + (0.6719*bilateral notch) + (0.0372 *age) + (0.4949*black ethnicity) + (1.5033*history PET) + (-1.2217*previous term live birth) + (0.0367* BMI) | LR | RF | NR |
| Papageorghiou et al., 2005 | 11–14 weeks | - | LR | NR | NR |
| Harrington et al., 1997 | 12 – 16 weeks | Score = -95.9 + 13.4757 x gestational age (weeks) - 0.5056 x [gestational age (weeks)]’ + 3.4891 x [ 1 if bilateral notches present, 0 if not] + 1.971 3 x mean (right and left) time-averaged velocity (Z score) - 1.4018 x mean peak systolic velocity (Z score) + 1-861 1 x mean uterine artery pulsatility index (Z score) + 0.7249 x umbilical artery pulsatility index (2 scores) | LR | Score chart rule | NR |
| **Classical-based prognostic models for early-onset pre-eclampsia** | | | | | |
| Tang et al., 2022 | 14–18 weeks | Y = (−15.10) + (0.0890∗BMI) + (−0.888 if Multiparous) + (0.0479∗Maternal Age) + (0.407∗ if Pre–gestational diabetes mellitus) + (0.319∗if Family history of hypertension) + (0.0233∗ MAP measured at 5–10 weeks) + (0.0503∗MAP measured at 11–13 weeks) + (0.00609∗uric acid test at 11–13 weeks). | LR | RF | NR |
| Serra et al., 2020 | 8 – 13^+6^ | Early-onset PE prior odds = 0.005025 * 0.646 * 1.47 * 0.71* 6.256 probability = odds/(1 þ odds), the early- *0.974 ¼ 0.02064429.  Early-onset PE posterior odds = 0:02064429 * 4:546 = 0:0938:  Early-onset PE posterior probability = 0:0938/ (1 + 0:0938) = 0:0858:  Early-onset PE posterior risk = 1/0:0858 = 1:12 | LR | RF | NR |
| Kumar et al., 2016 | 11 – 13^+6^ weeks | - | LR | NR | NR |
| Crovetto et al., 2015 | 11–13 weeks | Early PE [a priori and a posteriori risk = ey/(1 + ey)]:A priori risk=?5.177 + (2.383 if black ethnicity) ?(1.105 if nulliparous) + (3.543 if parous, previous PE) + (2.229 if chronic hypertension) + (2.201 if renal disease) | LR | RF | NR |
| Yliniemi et al., 2015 | 9 – 13^+6^ weeks | - | LR | RF | NR |
| Baschat et al., 2014 | First trimester | log-odds of PET = - 8.72 + 0.157 (if nulliparous) + 0.341 (for history of hypertension) + 0.635 (for history of prior PET) + 0.064 (per unit MAP) -0.186 (per unit of log PAPP-A MoM) | LR | RF | NR |
| Crovetto et al., 2014 | 11–13 weeks | - | LR | NR | NR |
| Scazzocchio et al., 2013 | 11-13^+6^ weeks | Priori risk for early-onset PE, Y= –7.703+(0.086 * BMI) + (1.708 if chronic hypertension) + (4.033 if renal disease) + (1.931 if parous, previous PE) + (0.005 if parous, no previous PE),  Posterior risk for early onset PE, Y = –0.320 + (2.681 * log a priori risk) + (13.132 * log MoM mean UtA-PI) + (25.733 * log MoM MAP) | LR | RF | NR |
| Parra‐Cordero et al., 2013 | 11-13^+6^ weeks | Early pre-eclampsia=−6.942+(0.074×BMI) + (1.878 if smoker) + (2.1116×log lowest UtA-PI MoM) – 0.671 * log PIGF MoM) | LR | RF | NR |
| Kuc et al., 2013 |  | Prior risk EO-PE=26.790–0.1196maternal height (cm)  +4.85656 Ln maternal weight +1.8456nulliparity [1] +0.0866 maternal age (years) +1.3536smoking | LR | RF | NR |
| Caradeux et al., 2013 | 11–14 weeks | Y = -4:4 + (-0.06*Age) + (-0.6*Multiparous) + (1.8*Previous PE) + (2.5*HT) + (-0.08) Weight) + (-1.7 *SBP) + (-3.3 *DBP) + (5.1*MAP) + (1.1*logUtaPI) + (0.9*Preterm labor) | LR | RF | NR |
| Di Lorenzo et al., 2012 | 11 -13 weeks | - | LR | NR | NR |
| Akolekar et al., 2011 | 11–13 weeks | - | LR | NR | NR |
| Kuijk et al., 2011 | - | LP = 0.29 - 0.42 *circulating level of glucose+ 0.59 *hypertension + 0.01*gestational age at delivery +0.41 * SGA * 0.01*BMI | LR | RF | NR |
| Poon et al., 2009 | 11–13 weeks | Y =−0.903 + (2.354 × log maternal factor-derived a-priori risk for early PE) +(11.194 × logUtA-PI (lowest) MoM) | LR | RF | NR |
| Poon et al., 2009 | 11–13 weeks | Y =−7.318 − 2.864 × log PAPP-A MoM + 11.456 × log UtA-PI MoM+ (1.939 if history of chronic hypertension)  + (1.569 if black, 0 if other racial origin) (0 if nulliparous − 0.869 if parous without previous PE, 1.297 if parous with previous PE) | LR | RF | NR |
| Poon et al., 2009 | 11–13 weeks | Y= -8.776 +14.177*log uterine artery PI MoM + 42.960*log MAP MoM - 2.249*log PAPP-A MoM - 3.529*log PlGF MoM + 0.120*BMI in kg/m2 + ( -1.472 if parous with no previous PE or 0 if nulliparous or parous with previous PE | LR | RF | NR |
| Akolekar et al., 2008 | 11-13^+6^ weeks | Y =−5.620 − (4.717 × log PlGF MoM) − (1.865 × log PAPP-A MoM) + (14.519 × uterine artery PI MoM) + (5.471 if history of chronic hypertension) + (1.159 if black, 0 if other racial origin) | LR | RF | NR |
| Plasencia et al., 2008 | 11-13^+6^ weeks | Y = −6.546 + (3.769 if the patient had chronic hypertension, 0 if she did not) + (15.692×  uterine artery PI (log MoM)) | LR | RF | NR |
| Onwudiwe et al., 2008 | 22–24 weeks | Log MoM UtA-PI = 0.2041 − (−0.0014 + 0.0132 for Log MoM MAP = 1.9542 − (1.8544 + 0.0025 × 25 for black ethnicity + 0 for non-smoker) = 0.1923  BMI + 0 for black ethnicity + 0 for nulliparity + 0for Y =−11.4487 + 31.2443 × 0.1923(log MoM UtA-PI) +spontaneous conception) = 0.0373  40.1105 × 0.0373 (log MoM MAP + 1.5442 (Afro-Caribbean) | LR | RF | NR |
| **Classical-based prognostic models for late-onset pre-eclampsia** | | | | | |
| Bunyapipat et al., 2023 | 15^+0^-20^+6^ weeks | Risk score = 12*Inhibin A ≥ 0.5–≤1 MoM + 20* Inhibin A > 1–≤2 MoM + 35*Inhibin A > 2 MoM + 10*Maternal age ≥ 35 + 12*History of preeclampsia + 37*History of infertile + 29*Cardiac disease + 34*Chronic hypertension + 21*Thyroid disease | LR | Score Chart rule | NR |
|  |  |  |  |  |  |
| Kumar et al., 2016 | 11 – 13^+6^ weeks | - | LR | NR | NR |
| Crovetto et al., 2015 | 11–13 weeks | Late PE [a priori and a posteriori risk = ey/(1 + ey)]:A priori risk=?5.177 + (2.383 if black ethnicity) ?(1.105 if nulliparous) + (3.543 if parous, previous PE) + (2.229 if chronic hypertension) + (2.201 if renal disease) | LR | RF | NR |
| Crovetto et al., 2014 | 11–13 weeks | - | LR | NR | NR |
| Scazzocchio et al., 2013 | 11 – 13^+6^ weeks | Late PE Y = –6.135 + (2.124 if previous PE) + (1.571 if chronic hypertension)  + (0.958 if diabetes mellitus) + (1.416 if thrombophilic condition) – (0.487 if multipara)  + (0.093 * BMI) | LR | RF | NR |
| Parra‐Cordero et al., 2013 | 11-13^+6^ weeks | Late pre-eclampsia=−5.584+(0.137×BMI) + (0.822×log lowest UtA-PI MoM) − (0.533×log PlGFMoM) | LR | RF | NR |
| Kuc et al., 2013 |  | Prior risk EO-PE=26.790–0.1196maternal height (cm)  +4.85656 Ln maternal weight +1.8456nulliparity [1] +0.0866 maternal age (years) +1.3536smoking | LR | RF | NR |
| Di Lorenzo et al., 2012 | 11–13 weeks | - | LR | NR | NR |
| Akolekar et al., 2011 | 11–13 weeks | - | LR | NR | NR |
| Youssef et al., 2011 | 11 – 13^+6^ weeks | - | LR | NR | NR |
| Poon et al., 2009 | 11–13 weeks | Y =−0.903 + (2.354 × log maternal factor-derived a-priori risk for early PE) +(11.194 × logUtA-PI (lowest) MoM) | LR | RF | NR |
| Poon et al., 2009 | 11–13 weeks | Y= -8.776 +14.177*log uterine artery PI MoM + 42.960*log MAP MoM - 2.249*log PAPP-A MoM - 3.529*log PlGF MoM + 0.120*BMI in kg/m2 + ( -1.472 if parous with no previous PE or 0 if nulliparous or parous with previous PE | LR | RF | NR |
| Poon et al., 2009 | 11–13 weeks | Y= -8.776 +14.177*log uterine artery PI MoM + 42.960*log MAP MoM - 2.249*log PAPP-A MoM - 3.529*log PlGF MoM + 0.120*BMI in kg/m2 + ( -1.472 if parous with no previous PE or 0 if nulliparous or parous with previous PE | LR | RF | NR |
| Akolekar et al., 2008 | 11–13 weeks | Y =−5.620 − (4.717 × log PlGF MoM) − (1.865 × log PAPP-A MoM) + (14.519 × uterine artery PI MoM) + (5.471 if history of chronic hypertension) + (1.159 if black, 0 if other racial origin) | LR | RF | NR |
| Plasencia et al., 2008 | 11–13 weeks | Y = −6.546 + (3.769 if the patient had chronic hypertension, 0 if she did not) + (15.692×  uterine artery PI (log MoM)) | LR | RF | NR |
| Onwudiwe et al., 2008 | 22–24 weeks | Log MoM UtA-PI = 0.2041 − (−0.0014 + 0.0132 for Log MoM MAP = 1.9542 − (1.8544 + 0.0025 × 25 for black ethnicity + 0 for non-smoker) = 0.1923 | LR | RF | NR |
| **Classical-based prognostic models for pre-term pre-eclampsia** | | | | | |
| Sandström *et al.*, 2021 | 14–17 weeks | - | LR | RF | NR |
| Tang *et al.*, 2022 | 11–13 weeks | Y = (−16.00) + (0.0586∗BMI) + (−0.968 if Multiparous) + (0.0445∗ Maternal Age) + (0.509∗ if Pre–gestational diabetes mellitus) + (0.455∗ if Family history of hypertension) + (0.0203∗ MAP measured at 5–10 weeks) + (0.110∗ MAP measured at 32– 35 weeks) + (0.00938∗ uric acid test at 32–35 weeks) + (−0.0751∗ alkaline phosphatase test at 32–35 weeks).  Period | LR | RF | NR |
| Pihl et al., 2020 | 11 weeks | - | LR | NR | NR |
| Sepúlveda‐Martínez *et al.*,2019[129] | 11 – 13^+6^ weeks | y = −5:354954 + maternal age × 0:0218775) + (1:534937 ½if chrHT) + (2:026845 [if SLE] + (0 [if nulliparous]) – (0:1196494 [if parous without previous PE) + (2:754261 [if parous with previous PE]) |  |  |  |
| Myers et al., 2013 | 14–16 weeks | Clinical risk factors: ?8.4093 + 0.9037 9 fertility treatment + 0.7999 9 any sister with pre-eclampsia + 0.1030 9 MAP; Clinical risk + 15 week PlGF MoM: ?7.7769 + 0.7307 9 fertility treatment + 0.1047 9 MAP? 1.7269 9 PlGF; Clinical risk + 20 week uterine Doppler: ?13.5946 + 0.8402 9 fertility treatment + 0.1039 9 MAP + 7.0938 9 20 week mean uterine artery RI; Clinical risk + 15 week PlGF MoM + uterine Doppler 20 week: ?12.5382 + 0.1078 9 MAP? 1.5658 9 PlGF + 6.1087 9 20 week mean uterine artery RI; Clinical risk + 15 week PlGF MoM + 20 week uterine Doppler + 20 week endoglin: ?10.4272 + 0.0994 9 MAP? 1.1787 9 PlGF + 0.0344 9 endoglin 20 week + 0.5285 9 20-week bilateral notches of uterine arteries.  1220 | LR | RF | NR |
| Akolekar et al., 2013 | 11–13 weeks | 55.0081 – 0.10367*age – 0.07259*weight +12.4007 - 3.0357*black – 1.770.9*South Asian -3.03*Parous with Pre-eclampsia + 3.14 * parous with no PE -1.188 * mother had PE -1.79*conception by vitro fertilisation - 4.0.6* SLE/APS – 6.24*chronic hypertension - 3.85*type 1 DM + 0.64*UtA-PI + 0.11*MAP -0.656 * PAPP-A + 0.86*PLGF | CRM | RF | NR |
| Wright et al., 2012 | 11–13 weeks | 55.0081 – 0.10367*age – 0.07259*weight +12.4007 - 3.0357*black – 1.770.9*South Asian -3.03*Parous with Pre-eclampsia + 3.14 * parous with no PE -1.188 * mother had PE -1.79*conception by vitro fertilisation - 4.0.6* SLE/APS – 6.24*chronic hypertension - 3.85*type 1 DM + 0.64*UtA-PI + 0.11*MAP | CRM | RF | NR |

NB: LR = Logistic Regression, CRM = Competing Risks Model, RF = Regression Formula, SLE = Systemic lupus erythematosus; APS = antiphospholipid syndrome, NR = Not Reported

Supplementary Table 3: Characteristics of early-onset pre-eclampsia prediction models.

| **S.No** | **Author, year** | **Country** | **Data sources** | **Centre** | **Events/Samples (events per predictor)** |
| --- | --- | --- | --- | --- | --- |
| 1 | Tang *et al.*, 2022 [70] | China | Retrospective cohort | Single | 46/20582 (7) |
| 2 | Serra *et al.*, 2020 [109] | Spain | Prospective cohort | Single | 161/6893 (18) |
| 3 | Kumar *et al.*, 2016 [86] | India | Prospective cohort | Single | -/3069 (-) |
| 4 | Crovetto *et al.*, 2015 [110] | France | Prospective cohort | Multicentre | 57/9462 (11) |
| 5 | Yliniemi *et al.*, 2015 [111] | Finland | Case-control | Single | 64/816 (8) |
| 6 | Baschat *et al.*, 2014 [90] | USA | Prospective cohort | Multicentre | 18/2441 (6) |
| 7 | Crovetto *et al.*, 2014 [112] | France | Nested case-control | Single | 28/5759 (5) |
| 8 | Scazzocchio et al., 2013 [113] | France | Prospective cohort | Single | 26/5170 (5) |
| 9 | Parra‐Cordero *et al.*, 2013 [114] | Chile | Nested case-control | Single | 17/359 (4) |
| 10 | Kuc et al., 2013 [115] | Netherlands | Nested case-control | Single | 68/500 (14) |
| 11 | Caradeux *et al.*, 2013 [116] | Chile | Prospective cohort | Multicentre | 29/627 (3) |
| 12 | Di Lorenzo *et al.*, 2012 [117] | Italy | Prospective cohort | Single | 12/2118 (4) |
| 13 | Akolekar *et al.*, 2011 [118] | UK | Prospective cohort | Single | 112/33602 (12) |
| 14 | Kuijk et al., 2011 [119] | Netherlands | Prospective cohort | Multicentre | 28/407 (6) |
| 15 | Poon *et al.*, 2009 [120] | UK | Prospective cohort | Single | 37/8061 (6) |
| 16 | Poon *et al.*, 2009 [121] | UK | Prospective cohort | Single | 32/8051 (6) |
| 17 | Poon *et al.*, 2009 [122] | UK | Prospective cohort | Single | 34/7797 (5) |
| 18 | Akolekar et al., 2008 [123] | UK | Case-control | Single | 29/824 (5) |
| 19 | Plasencia et al., 2008 [124] | Denmark | Prospective cohort | Single | 22/3107 (11) |
| 20 | Onwudiwe *et al.*, 2008 [125] | UK | Routinely Collected | Single | 23/3347 (8) |

Supplementary Table 4: Characteristics of late-onset pre-eclampsia prediction models.

| **S.No** | **Author, year** | **Country** | **Data sources** | **Centre** | **Events/Samples (events per predictor)** |
| --- | --- | --- | --- | --- | --- |
| 1 | Bunyapipat *et al.*, 2023 [67] | Thailand | Retrospective cohort | Single | 55/2000 (8) |
| 2 | Kumar *et al.*, 2016 [86] | India | Prospective cohort | Single | NR/3069 (NR) |
| 3 | Crovetto *et al.*, 2015 [110] | France | Prospective cohort | Multicentre | 246/9462 (41) |
| 4 | Crovetto *et al.*, 2014 [112] | France | Nested case-control | Single | 84/5759 (14) |
| 5 | Scazzocchio et al., 2013 [113] | France | Prospective cohort | Single | 110/5170 (18) |
| 6 | Parra‐Cordero *et al.*, 2013 [114] | Chile | Nested case-control | Single | 53/359 (18) |
| 7 | Kuc et al., 2013 [115] | Netherlands | Nested case-control | Single | 99/500 (17) |
| 8 | Di Lorenzo *et al.*, 2012 [117] | Italy | Prospective cohort | Single | 13/2118 (4) |
| 9 | Akolekar *et al.*, 2011 [118] | UK | Prospective cohort | Single | 453/33602 (75) |
| 10 | Youssef *et al.*, 2011 [126] | Italy | Prospective cohort | Single | 13/528 (4) |
| 11 | Poon *et al.*, 2009 [120] | UK | Prospective cohort | Single | 128/8061 (21) |
| 12 | Poon *et al.*, 2009 [121] | UK | Prospective cohort | Single | 124/8051 (21) |
| 13 | Poon *et al.*, 2009 [122] | UK | Prospective cohort | Single | 123/7797 (18) |
| 14 | Akolekar et al., 2008 [123] | UK | Case-control | Single | 98/824 (9) |
| 15 | Plasencia et al., 2008 [124] | Denmark | Prospective cohort | Single | 71/3107 (11) |
| 16 | Onwudiwe *et al.*, 2008 [125] | UK | Routinely Collected | Single | 78/3347 (13) |

Supplementary Table 5: Characteristics of preterm pre-eclampsia prediction models.

| **S.No** | **Author, year** | **Country** | **Data sources** | **Centre** | **Events/Samples (events per predictor)** |
| --- | --- | --- | --- | --- | --- |
| 1 | Sandström *et al.*, 2021 [150] | Sweden | Prospective cohort | Single | 2576/58899 (258) |
| 2 | Tang *et al.*, 2022 [70] | China | Retrospective cohort | Single | 119/20582 (17) |
| 3 | Pihl et al., 2020 [128] | Denmark | Case-control | Single | 55/717 (13) |
| 4 | Sepúlveda‐Martínez *et al.*,2019 [129] | Chile | Case-control | Single | 49/1756 (3) |
| 5 | Myers et al., 2013 [130] | SCOPE^a^ | Prospective cohort | Multicentre | 187/3529 (NR) |
| 6 | Akolekar et al., 2013 [15] | UK | Prospective cohort | Multicentre | 568/5884 (44) |
| 7 | Wright et al., 2012 [131] | UK | Prospective cohort | Multicentre | 568/5884 (51) |

NB: ^a^ = New Zealand, Australia, the UK, and Ireland.

Supplementary Table 6: Model performance measures in any-, early- and late-onset, and preterm pre-eclampsia classical regression studies.

| **S.No** | **Developed study** | **Country** | **Events /Samples (events per predictors)** | **Model discrimination performance** | **Model calibration performance** | **Model validation:**  **Internal or External** | **Deployment strategies** |
| --- | --- | --- | --- | --- | --- | --- | --- |
| **Model performance measures in any-onset pre-eclampsia classical regression studies** | | | | | | | |
| 1 | Suksai et al., 2022 [68] | Thailand | 167/4600 (14) | 0.77 (0.73 – 0.81) |  |  | Risk score |
| 2 | Tarca *et al.*, 2022 [69] | USA | 166/1150 (42) | 0.70 (0.66 – 0.74) |  |  | Regression formula |
| 3 | Tang *et al.*, 2022 [70] | China | 717/20582 (102) | 0.74 (NR) |  | Internal, split-sample |  |
| 4 | Yue *et al.*, 2021 [71] | China | 310/6064 (34) | 0.86 (0.84 – 0.88) | Well calibrated* | Internal, split-sample |  |
| 5 | Kim et al., 2021 [72] | China | 13/351 (4) | 0.83 (NR) |  |  |  |
| 6 | Wang et al., 2020 [73] | China | 25/356 (6) | 0.88 (NR) |  | Internal, 10-fold cross validation |  |
| 7 | Al-Rubaie *et al.*, 2020 [74] | Australia | 293/12395 (37) | 0.70 (0.66 – 0.73) | Slope = 0.44 | Internal, bootstrap | Regression formula |
| 8 | Sovio and Smith, 2019 [75] | UK | 28/4184 (3) | 0.85 (0.79 – 0.91) |  | Externally validated | Regression formula |
| 9 | Boutin *et al.*, 2019 [76] | Canada | 232/4739 (38) | 0.66 (0.62 – 0.71) |  |  |  |
| 10 | Boutin *et al.*, 2018 [77] | Canada | 232/4665 (33) | 0.62 (0.58 – 0.66) |  |  |  |
| 11 | Cheng *et al.*, 2018 [78] | China | 30/3330 (10) | 0.72 (0.64 – 0.81) |  |  | Regression formula |
| 12 | [Da Silva Costa](https://research.monash.edu/en/persons/fabricio-da-silva-costa) F *et al.*, 2018 [79] | Brazil | 40/372 (10) | 0.70 (NR) |  |  |  |
| 13 | Asiltas et al., 2018 [80] | Turkey | 38/160 (12) | 0.91 (0.86 – 0.95) |  |  |  |
| 14 | Rocha *et al.*, 2017 [81] | Brazil | 55/733 (14) | 0.79 (0.76 – 0.82) |  |  | Regression formula |
| 15 | Luo and Han, 2017 [82] | China | 33/104 (11) | 0.81 (0.73 – 0.89) |  |  |  |
| 16 | Agarwal et al., 2017 [83] | India | 35/291(11) | 0.96 (0.92 – 1) |  |  |  |
| 17 | Guy *et al.*, 2017 [84] | UK | 66/2764 (5) | 0.88 (0.85 – 0.92) |  |  |  |
| 18 | Gabbay‐Benziv., 2016 [85] | USA | 108/2433 (21) | 0.78 (0.78 – 0.85) |  |  | Regression formula |
| 19 | Kumar *et al.*, 2016 [86] | India | 98/3069 (20) | 0.73 (NR) |  |  |  |
| 20 | Giguere *et al.*, 2015 [87] | Canada | 96/343 (16) | 0.77 (0.70 – 0.82) |  | Externally validated |  |
| 21 | Wright et al., 2015 [88] | UK | 2704/120492 (540) | 0.76 (NR) |  | Internal, 5-fold cross validation, externally validated |  |
| 22 | Moon and Odibo, 2015 [89] | USA | 102/1177 (15) | 0.84 (0.75 – 0.91) |  |  |  |
| 23 | Baschat *et al.*, 2014 [90] | USA | 108/2441 (27) | 0.82 (0.78 – 0.86) |  | Internal, 10-fold cross validation, Externally validated | Regression formula |
| 24 | Kenny *et al.*, 2014 [91] | SCOPE^a^ | 278/5623 (56) | 0.68 (0.63 – 0.74) |  | Internal, split-sample, externally validated |  |
| 25 | Goetzinger *et al.*, 2014 [92] | USA | 49/578(8) | 0.76 (0.69 – 0.83) |  | Internal, split-sample | Risk score |
| 26 | Gurgel Alves *et al.*, 2014 [93] | Brazil | 31/550 (6) | 0.83 (NR) |  |  |  |
| 27 | Teixeira *et al.*, 2014 [94] | Portugal | 140/4799 (12) | 0.73 (NR) |  |  |  |
| 28 | Skråstad *et al.*, 2014 [95] | Norway | 39/640 (10) | 0.87 (0.76 – 0.98) |  |  |  |
| 29 | Direkvand-M et al., 2013 [96] | Iran | 58/610 (11) | 0.67 (0.59 – 0.67) |  |  | Regression formula |
| 30 | North *et al.*, 2011 [97] | SCOPE^a^ | 186/3529 (16) | 0.77 (0.75 – 0.80) | Not calibrated | Internal, 10-fold cross validation, externally validated | Regression formula |
| 31 | Odibo *et al.*, 2011 [98] | USA | 42/452 (14) | 0.77 (0.63 – 0.81) |  | Externally validated | Regression formula |
| 32 | Yu *et al.*, 2011 [99] | China | 31/124 (7) | 0.94 (0.89 – 0.99) |  |  |  |
| 33 | Goetzinger *et al.*, 2010 [100] | USA | 293/3716 (59) | 0.70 (0.65 – 0.72) |  | Externally validated | Risk score |
| 34 | Thilaganathan *et al.*, 2010 [101] | UK | 45/170 (15) | 0.83 (0.74 – 0.91) |  |  |  |
| 35 | Poon *et al.*, 2008 [102] | UK | 104/5193 (26) | 0.80 (NR) |  | Externally validated | Regression formula |
| 36 | Deis *et al.*, 2008 [103] | France | 110/4777 (18) | 0.74 (NR) | Well calibrated | Internal, bootstrap | Nomogram |
| 37 | De Paco *et al.*, 2008 [104] | UK | 83/4617 (17) | 0.81 (0.77 – 0.86) |  |  |  |
| 38 | Pilalis *et al.*, 2007 [105] | Greece | 13/878 (2) | 0.75 (0.72 – 0.78) |  |  |  |
| 39 | Yu et al., 2005 [106] | UK | 612/30708 (76) | 0.83 (NR) |  | Internal, split-sample, externally validated | Regression formula |
| 40 | Papageorghiou et al., 2005 [107] | UK | 369/17480 (53) | 0.79 (NR) |  |  |  |
| 41 | Harrington *et al.*, 1997 [108] | UK | 30/626 (7) | NR |  |  |  |
| **Model performance measures in early-onset pre-eclampsia classical regression studies** | | | | | | | |
| 1 | Tang *et al.*, 2022 [70] | China | 46/20582 (7) | 0.78 (NR) |  |  |  |
| 2 | Serra *et al.*, 2020 [109] | Spain | 161/6893 (18) | 0.98 (0.97 – 0.97) |  |  | Regression formula |
| 3 | Kumar *et al.*, 2016 [86] | India | -/3069 (-) | NR |  |  |  |
| 4 | Crovetto *et al.*, 2015 [110] | France | 57/9462 (11) | 0.96 (0.92 – 0.99) |  | Externally validated |  |
| 5 | Yliniemi *et al.*, 2015 [111] | Finland | 64/816 (8) | 0.79 (0.73 – 0.85) |  |  | Risk score |
| 6 | Baschat *et al.*, 2014 [90] | USA | 18/2441 (6) | 0.83 (0.74 – 0.91) |  | Internal, 10-fold cross validation, externally validated | Regression formula |
| 7 | Crovetto *et al.*, 2014 [112] | France | 28/5759 (5) | 0.96 (0.92 – 0.99) |  |  |  |
| 8 | Scazzocchio et al., 2013 [113] | France | 26/5170 (5) | 0.95 (0.94 – 0.98) |  | Externally validated | Regression formula |
| 9 | Parra‐Cordero *et al.*, 2013 [114] | Chile | 17/359 (4) | 0.85 (-) |  | Externally validated | Regression formula |
| 10 | Kuc et al., 2013 [115] | Netherlands | 68/500 (14) | 0.88 (-) |  | Externally validated | Regression formula |
| 11 | Caradeux *et al.*, 2013 [116] | Chile | 29/627 (3) | 0.89 (-) |  | Externally validated | Regression formula |
| 12 | Di Lorenzo *et al.*, 2012 [117] | Italy | 12/2118 (4) | 0.89 (-) |  | Externally validated |  |
| 13 | Akolekar *et al.*, 2011 [118] | UK | 112/33602 (12) | NR |  |  | Regression formula |
| 14 | Kuijk et al., 2011 [119] | Netherlands | 28/407 (6) | 0.65 (0.56 – 0.74) | Well calibrated |  |  |
| 15 | Poon *et al.*, 2009 [120] | UK | 37/8061 (6) | 0.81 (NR) |  | Externally validated | Regression formula |
| 16 | Poon *et al.*, 2009 [121] | UK | 32/8051 (6) | 0.90 (NR) |  | Externally validated | Regression formula |
| 17 | Poon *et al.*, 2009 [122] | UK | 34/7797 (5) | 0.91 (0.86 – 0.96) |  | Externally validated | Regression formula |
| 18 | Akolekar et al., 2008 [123] | UK | 29/824 (5) | 0.94 (0.89 – 0.99) |  | Externally validated | Regression formula |
| 19 | Plasencia et al., 2008 [124] | Denmark | 22/3107 (11) | 0.91 (0.90 – 0.91) |  | Externally validated | Regression formula |
| 20 | Onwudiwe *et al.*, 2008 [125] | UK | 23/3347 (8) | 0.99 (0.99 - 1) |  |  | Regression formula |
| **Model performance measures in late-onset pre-eclampsia classical regression studies** | | | | | | | |
| 1 | Bunyapipat *et al.*, 2023 [67] | Thailand | 55/200 (8) | 0.78 (NR) |  |  |  |
| 2 | Kumar *et al.*, 2016 [86] | India | -/3069 (-) | NR |  |  |  |
| 3 | Crovetto *et al.*, 2015 [110] | France | 246/9462 (41) | 0.87 (0.84 – 0.90) |  | Externally validated | regression formula |
| 4 | Crovetto *et al.*, 2014 [112] | France | 84/5759 (14) | 0.89 (0.84 – 0.94) |  |  |  |
| 5 | Scazzocchio et al., 2013 [113] | France | 110/5170 (18) | 0.71 (0.66 – 0.76) |  | Externally validated |  |
| 6 | Parra‐Cordero *et al.*, 2013 [114] | Chile | 53/359 (18) | NR |  | Externally validated | Regression formula |
| 7 | Kuc et al., 2013 [115] | Netherlands | 99/500 (17) | 0.85 (-) |  | Externally validated | Regression formula |
| 8 | Di Lorenzo *et al.*, 2012 [117] | Italy | 13/2118 (4) | 0.89 (-) |  | Externally validated |  |
| 9 | Akolekar *et al.*, 2011 [118] | UK | 453/33602 (75) | NR |  |  | Regression formula |
| 10 | Youssef *et al.*, 2011 [126] | Italy | 13/528 (4) | 0.82 (0.64 – 0.99) |  |  |  |
| 11 | Poon *et al.*, 2009 [120] | UK | 128/8061 (21) | NR |  | Externally validated | Regression formula |
| 12 | Poon *et al.*, 2009 [121] | UK | 124/8051 (21) | 0.80 (NR) |  | Externally validated | Regression formula |
| 13 | Poon *et al.*, 2009 [122] | UK | 123/7797 (18) | 0.81 (0.78 – 0.85) |  | Externally validated | Regression formula |
| 14 | Akolekar et al., 2008 [123] | UK | 98/824 (9) | 0.81 (0.77 – 0.86) |  |  | Regression formula |
| 15 | Plasencia et al., 2008 [124] | Denmark | 71/3107 (11) | 0.82 (0.82 – 0.83) |  | Externally validated | Regression formula |
| 16 | Onwudiwe *et al.*, 2008 [125] | UK | 78/3347 (13) | 0.84 (0.82 – 0.85) |  |  | Regression formula |
| **Model performance measures in preterm pre-eclampsia classical regression studies** | | | | | | | |
| 1 | Sandström *et al.*, 2022 [127] | Sweden | 2576/58899 (258) | 0.87 (0.84 – 0.90) |  |  |  |
| 2 | Tang *et al.*, 2022 [70] | China | 119/20582 (17) | 0.78 (NR) |  |  |  |
| 3 | Pihl et al., 2020 [128] | Denmark | 55/717 (13) | 0.74 (NR) |  |  |  |
| 4 | Sepúlveda‐Martínez *et al.*,2019 [129] | Chile | 49/1756 (3) | 0.89 (0.84 -0.96) |  |  |  |
| 5 | Myers et al., 2013 [130] | SCOPE^a^ | 187/3529 (-) | 0.76 (0.67 – 0.84) |  |  |  |
| 6 | Akolekar et al., 2013 [15] | UK | 568/5884 (44) | NR |  | Externally validated |  |
| 7 | Wright et al., 2012 [131] | UK | 568/5884 (51) | NR |  | Externally validated |  |

NB: * == the celebration status reported in the article but not reported the metrics values, ^a^ = New Zealand, Australia, the UK, and Ireland.
